# The Crosstalk between Nrf2 and TGF-β1 in the Epithelial-Mesenchymal Transition of Pancreatic Duct Epithelial Cells

**DOI:** 10.1371/journal.pone.0132978

**Published:** 2015-07-30

**Authors:** Sarah Arfmann-Knübel, Birte Struck, Geeske Genrich, Ole Helm, Bence Sipos, Susanne Sebens, Heiner Schäfer

**Affiliations:** 1 Laboratory of Molecular Gastroenterology, Dept. of Internal Medicine I, UKSH Campus Kiel, Arnold-Heller-Str. 3, Bldg. 6, 24105, Kiel, Germany; 2 Group Inflammatory Carcinogenesis, Institute of Experimental Medicine, CAU Kiel, Arnold-Heller-Str. 3, Bldg. 17, 24105, Kiel, Germany; 3 Department of Pathology and Neuropathology, University Hospital Tübingen, Liebermeisterstraße 8, 72076, Tübingen, Germany; Northwestern University, UNITED STATES

## Abstract

Nrf2 and TGF-β1 both affect tumorigenesis in a dual fashion, either by preventing carcinogen induced carcinogenesis and suppressing tumor growth, respectively, or by conferring cytoprotection and invasiveness to tumor cells during malignant transformation. Given the involvement of Nrf2 and TGF-β1 in the adaptation of epithelial cells to persistent inflammatory stress, e.g. of the pancreatic duct epithelium during chronic pancreatitis, a crosstalk between Nrf2 and TGF-β1 can be envisaged. By using premalignant human pancreatic duct cells (HPDE) and the pancreatic ductal adenocarcinoma cell line Colo357, we could show that Nrf2 and TGF-β1 independently but additively conferred an invasive phenotype to HPDE cells, whereas acting synergistically in Colo357 cells. This was accompanied by differential regulation of EMT markers like vimentin, Slug, L1CAM and E-cadherin. Nrf2 activation suppressed E-cadherin expression through an as yet unidentified ARE related site in the E-cadherin promoter, attenuated TGF-β1 induced Smad2/3-activity and enhanced JNK-signaling. In Colo357 cells, TGF-β1 itself was capable of inducing Nrf2 whereas in HPDE cells TGF-β1 per-se did not affect Nrf2 activity, but enhanced Nrf2 induction by tBHQ. In Colo357, but not in HPDE cells, the effects of TGF-β1 on invasion were sensitive to Nrf2 knock-down. In both cell lines, E-cadherin re-expression inhibited the proinvasive effect of Nrf2. Thus, the increased invasion of both cell lines relates to the Nrf2-dependent downregulation of E-cadherin expression. In line, immunohistochemistry analysis of human pancreatic intraepithelial neoplasias in pancreatic tissues from chronic pancreatitis patients revealed strong Nrf2 activity already in premalignant epithelial duct cells, accompanied by partial loss of E-cadherin expression. Our findings indicate that Nrf2 and TGF-β1 both contribute to malignant transformation through distinct EMT related mechanisms accounting for an invasive phenotype. Provided a crosstalk between both pathways, Nrf2 and TGF-β1 mutually promote their tumorigenic potential, a condition manifesting already at an early stage during inflammation induced carcinogenesis of the pancreas.

## Introduction

The antioxidant transcription factor Nuclear factor E2 related factor-2 (Nrf2) and the cytokine Transforming growth factor beta1 (TGF-β1) both have a dual role in carcinogenesis [1–8]. Nrf2 primarily confers cytoprotection by regulating the cellular response to xenobiotic and oxidative stress [9,10]. By inducing a battery of cytoprotective and detoxifying or antioxidant enzymes, Nrf2 mitigates stress induced damage of cell components like DNA and thus prevents mutagenesis. Based on these effects, Nrf2 was regarded as being anti-tumorigenic and to serve as beneficial target in chemoprevention [10–12]. However, due to its capability to confer greater survival to cells along with persistent stress adaptation, an amplified Nrf2 activity has been shown to favour tumorigenesis, as well [6,13,14]. This is underlined by the fact that tumors frequently associate with deregulated Nrf2 activation, resulting from various genetic and epigenetic alterations [4–7,15], from persistent oxidative stress–e.g. during chronic inflammation [15,16]—or from the interplay with Nrf2 and oncogenic pathways [4–7,15,17]. The growth advantages of cancer cells depending on Nrf2 include apoptosis protection and chemo-/radioresistance [18–20], increased proliferation rates [21,22], greater invasiveness [23,24] and angiogenesis [25,26], and an altered metabolism [27,28].

TGF-β1, on the one hand, is also known to potentially inhibit tumor growth by impeding cell cycle progression of transformed cells [3,29]. On the other hand, TGF-β1 has a crucial role in malignant transformation by conferring invasive properties to cancer cells, particularly through the process of epithelial mesenchymal transition (EMT) taking place in many types of adenocarcinomas [3,29]. Both, Nrf2 [17,21,30] and TGF-β1 [2,31,32] have been shown to be involved in the development of pancreatic ductal adenocarcinoma (PDAC) and to confer malignant properties already to untransformed pancreatic ductal epithelial cells [33–35]. This may relate to early events during pancreatic carcinogenesis that could arise from chronic inflammation.

Given that both Nrf2 [15,16,36] and TGF-β1 [31,37] are upregulated during persistent inflammation in order to mitigate oxidative damage and to suppress inflammatory events, these two mediators may act together quite early during inflammation associated carcinogenesis–e.g of the pancreas. A line of experimental data have shown that Nrf2 and TGF-β1 can inhibit each other. For instance, TGF-β1 has been demonstrated to suppress Nrf2 dependent expression of its antioxidant enzyme target NQO1 during liver fibrosis accounting for increased ROS level [38,39]. Conversely, Nrf2 inhibits the profibrotic action of TGF-β1 by blocking Smad3 activation [40,41], and Nrf2 has been shown to suppress promigratory signals in HepG2 hepatoma or A549 NSCLC cells [42].

In contrast, Nrf2 is able to promote a migratory phenotype in gallbladder carcinoma, esophagial squamous carcinoma or glioma cells [24,43,44] and thereby supports metastasis formation [26]. Moreover, through the downregulation of E-cadherin along with TGF-β1 induced EMT, E-cadherin dependent Nrf2-inhibition [45] may be abrogated resulting in an increased Nrf2 activity, whereas TGF-β1 may indirectly suppress Nrf2 activation through an elevated expression of Caveolin-1 known to inhibit Nrf2 [46]. Thus, the crosstalk between Nrf2 and TGF-β1 regarding EMT and malignant transformation is complex, but the modalities by which Nrf2 and TGF-β1 negatively or positively affect each other are not well known.

The present study investigated the mutual effects of Nrf2 activation and TGF-β1 on the phenotype of non-malignant pancreatic duct epithelial cells as well as PDAC cells. Surprisingly, we observed an increased invasive phenotype through TGF-β1 and Nrf2 which occurred in an additive way and depended on distinct mechanisms. These include potent downregulation of E-cadherin expression by Nrf2 and the shaping of TGF-β1 signalling from Smad dependent towards Smad independent pathways.

The findings point to a substantial cooperation between Nrf2 and TGF-β1 during initiation of pancreatic tumorigenesis, providing the rational of reconsidering current concepts for chemoprevention and cancer therapy.

## Materials and Methods

### Cell culture

The human pancreatic ductal epithelial cell line HPDE (kindly provided by M.S. Tsao, Ontario Cancer Center, Toronto, Canada) [47] was cultured as described [35]. The human PDAC cell line Colo357 [48] harboring wild-type Smad4/DPC4 (donated by H. Kalthoff, Experimental Cancer Research Institute, UKSH-Campus Kiel) was cultured in RPMI 1640 containing 10% FCS, 1% L-glutamine and 1% sodium pyruvate (all from PAA-Laboratories, Cölbe, Germany) [35]. Cells were cultured at 37°C, 5% CO_2_ and 85% humidity.

### Wound-healing assay

HPDE cells were grown to confluency in a 35 mm two chamber μ-Dish (Ibidi, Planegg, Germany). Then, cells were either left untreated or treated with 50 μM tBHQ or 10 ng/mL TGF-β1 and the insert was carefully removed forming a wound of 500 μm diameter. Images were taken at 0h, 8h and 24h and a 100× magnification. A uniform closure of the wound indicates continuation of proliferation whereas accumulation of single elongated cells within the gap is indicative for cell migration.

### Collagen invasion (Boyden Chamber) assay

Collagen I (Sigma-Aldrich, München, Germany) was coated (400 μg/mL, 120 minutes) on the backside of transwell inserts (24-well plate, 8 μm pore size from Greiner). Five x10^4^ cells were seeded into the upper chamber in 500 μL of the respective medium containing 1% FCS. Cells were allowed to invade into the lower compartment containing 500 μL medium (+1% FCS) for 24 hours at 37°C. To quantify invaded cells, transwells were removed and cleaned with a cotton swab to remove noninvaded cells. Invaded cells adherent to the bottom of the membrane were stained with crystal violet (Sigma-Aldrich). After washing in water, the remaining dye was eluted with 10% acidic acid and measured at 595 nm in an Opsys MR microplate reader (Dynex). Each determination was done in duplicates. To account for different proliferation rates, the total amount of seeded (non-invaded + invaded) cells was stained as described above and used for normalization. Data are expressed as OD migrated cells x 100/OD total cells (%).

### Westernblot

Nuclear and cytosolic extracts or total cell lysates were prepared as described before [49]. After electrophoresis and semi-dry electroblotting onto PVDF membranes, the following primary antibodies were used for immunodetection at 1:1000-fold dilution in 5% (w/v) non-fat milk powder, 0.05% Tween20 in TBS (Tris-buffered saline; 50 mM Tris/HCl, pH 7.6, and 150 mM NaCl): Nrf2, Slug, lamin-A/C, Smad2/3, vimentin and Hsp90 (all from Santa Cruz Biotechnology, Heidelberg, Germany), E-cadherin and JNK (both from Cell Signaling, Frankfurt/a.M., Germany) or L1 (clone L1-9.3, provided by Gerd Moldenhauer, DKFZ Heidelberg). In addition, the following antibodies diluted at 1:500 in in 5% (w/v) bovine serum albumin, 0.05% Tween20 in TBS were used: phospho-JNK, phospho Smad2 (Ser465/467) and phospho Smad3 (Ser423/425) (all from Cell Signaling). After incubation overnight at 4°C, blots were exposed to the appropriate horse-radish peroxidase-conjugated secondary antibody (Santa Cruz) diluted (1:1000) in blocking buffer and developed using the Dura detection kit (Perbio Sciences, Bonn, Germany). Data acquisition was done with the Chemidoc-XRS gel documentation system (BioRad, Munich, Germany) using the Quantity One software (Bio-Rad). Hsp90 and lamin-A/C served as loading control for total cell lysates and nuclear extracts, respectively.

### RNA preparation and RT-qPCR

Total RNA was isolated (peqGOLD total RNA kit, PeqLab, Erlangen, Germany) and subjected to reversed transcription (Fermentas, St.Leon-Rot, Germany) according to the manufacturer’s instructions. All primer were purchased from MWG/Eurofins (Munich, Germany). The follwing forward/reversed primers were used: *L1*, 5’-GAACTGGATGTGGTGGACAG-3’/5’-GAGGGTGGTAGAGGTCTGGT-3’; *TBP*, 5’-TATAATCCCAAGCGGTTTGC-3’/5’-GCTGGAAAACCCAACTTCTG-3’; *E-cadherin*, 5’- TGCTCTTGCTGTTTCTTCGG-3’/5’-TGCCCCATTCGTTCAAGTAG-3’; *vimentin*, 5’-TC CAAGTTTGCTGACCTCTC-3’/5’-TCAACGGCAAAGTTCTCTTC-3’; *slug*, 5’-ACACAC ACACACCCACAGAG-3’/5’-GCAAATGCTCTGTTGCAGTGA-3’; *NQO1*, 5’-AAATCCT GGAAGGATGGAAG–3’/5’-TTGTCAGTTGGGATGGACTT–3’; *GCLC*, 5’-CTGGGGAG TGATTTCTGCAT-3’/5’-AGGAGGGGCTTAAATCTCA-3’. The reaction was carried out with the MyiQ Single Color Real-time PCR Detection System (Bio-Rad, Munich, Germany) and fluorescence data were converted into cycle threshold (C_T_) measurements.

### siRNA treatment and cDNA transfection

For siRNA transfection, cells grown in 12-well plates were submitted to lipofection using 6 μl of the HiperFect reagent (Qiagen, Hilden, Germany) and 150 ng/well of either negative control siRNA (Qiagen) or Nrf2 siRNA (SI03246614, Qiagen). After 8 hours, medium was changed and cells were subject to further mono- or coculture. For transfection of E-cadherin cDNA, cells grown in 12-well plates were submitted to lipofection using the Effectene reagent (Qiagen) and 0.4 μg of a pcDNA3.1-E-cadherin vector (kindly provided by H. Ungefroren, Lübeck, Germany). As control, an empty pcDNA3.1 vector (Life-Technologies) was used. After overnight transfection, medium was replaced and cell culture continued for 8–24h. Then, cells were further processed as specified.

### Generation of E-cadherin promoter luciferase reporter gene constructs

Genomic DNA from HPDE cells was used as template for generating amplicons (forward primer 5’-aggtggtcctgacctagggaat-3’ / reversed primer: 5’-agtggcgtcggaactgcaaagctt-3’ and 5’-ctaacccatgaagctctacagtt-3’ / reversed primer: 5’-agtggcgtcggaactgcaaagctt-3’) consisting of 5’-parts of the human E-cadherin gene (position -1189 to +11 and -1153 to +11, respectively; gene bank accession no. DQ090940.1). After subcloning into the TOPO-pCR2.1 vector (Invitrogen), the promoter fragments were cloned into the pGL3 basic luciferase vector (Promega, Mannheim, Germany) via KpnI and HindIII sites. The constructs Ecad[–1189]-pGL3 and Ecad[–1153]-pGL3 containing and lacking the potential ARE site (pos. -1153 to -1162), respectively, were verified by automated BigDye (PerkinElmer, Waltham, USA) DNA cycle sequencing using an ABI 3770 instrument (PerkinElmer).

### Luciferase assays

For detecting Nrf2 activity, 0.2 μg p-ARE/ptkRL or the control vector/ptk-RL (all vectors from Qiagen/SABioscience) were transfected into cells grown in 12-well plates using Effectene (Qiagen). For detection of Smad activity, 0.3 μg p6SRE (containing six Smad binding elements [50] or 0.3 μg pGL3 (Promega) as control were transfected together with 0.1 μg ptkRL. For analysing E-cadherin promoter activity, 0.3 μg of Ecad[–1189]-pGL3, Ecad[–1153]-pGL3 or 0.3 μg pGL3 were transfected together with 0.1 μg ptkRL. After 8 hours, medium was changed and cells were cultured overnight followed by treatments as indicated. Afterwards, cells were washed with PBS and lysed in 150 μl/well passive lysis buffer (Promega, Mannheim, Germany). Lysates were centrifuged for 2 min at 4°C, 13000 rpm. Twenty μl of the supernatant were used for the dual luciferase assay procedure, using Dual-Glow luciferase assay system from Promega. Measurements were performed with a Berthold luminometer and firefly luciferase expression was normalized to constitutive renilla luciferase expression. All measurements were carried out in duplicates.

### Patients & tissues

Pancreatic tissues were obtained from CP patients during surgery and their conservation and histopathological diagnosis were performed at the Institute of Pathology, University Tübingen.

### Ethics statement

The research was approved by the ethics committee of the University Hospital Tübingen (reference 470/210BO1). Written informed consent was obtained from all patients.

### Immunohistochemistry

Consecutive sections (3 μm) of formalin fixed and paraffin embedded tissues from 20 CP patients were used for immunostaining of Ser40-P-Nrf2 (1:200, rabbit moAb, Abcam) indicating its activated state and of E-cadherin (1:100, mouse moAb, Cell Signaling). Isotype matched control antibody stainings were conducted in parallel using identical dilutions in 1% BSA and 0.3% Triton-X in PBS. Prior to primary antibody incubation (overnight, 4°C), sections were deparaffinized and rehydrated as described [51], then washed in PBS for 10 minutes and submitted to antigen retrieval by incubating in 1:10 diluted citrate buffer at pH 6.0 for 20 minutes at 95°C. Blocking was conducted by incubating in PBS supplemented with 4% BSA for 30 min at room temperature (RT). After primary antibody incubation, sections were washed three times at RT. To detect P-Nrf2 staining sections were incubated then (30 min, RT) with the Envision+ HRP anti rabbit reagent (Dako, Hamburg, FRG). To detect E-cadherin, sections were incubated (30 min, RT) with a biotin-conjugated secondary antibody anti mouse IgG (Jackson ImmunoResearch via Dianova, Hamburg, FRG) diluted in PBS + 0.3% Triton-X100, washed and reincubated (30 min, RT) with peroxidase conjugated streptavidin (Jackson ImmunoResearch via Dianova) diluted 1:500 in PBS + 0.3% Triton-X 100. Substrate reaction was performed with AEC Substrate (Dako) for 10 min at RT followed by washing in PBS. Then, sections were stained in Mayer`s Haemalaun (AppliChem, Darmstadt, Germany) for 2 min, washed in water for 10 min and finally covered with Kaiser`s glycerine gelatine (Waldeck, Munster, Germany). Usage of the isotype matched control antibodies revealed no or only weak background staining. The staining intensity and frequency in selected areas was scored as follows: no staining = score 0, faint staining in <33% = score 0.5, intense staining in <33% of the cells = score 1.0, intense staining in 33%- 66% of cells = score 1.5, intense staining in >66% of cells = score 2.0.

### Statistical analysis

Data are presented as mean ± standard deviation and were analyzed by using Excell 2013 Software (run on Microsoft Windows 8.1). A p-value < 0.05 determined by the student’s t-test was considered statistically significant.

## Results

### Nrf2 activity in premalignant and malignant pancreatic duct cells

**Fig 1:**
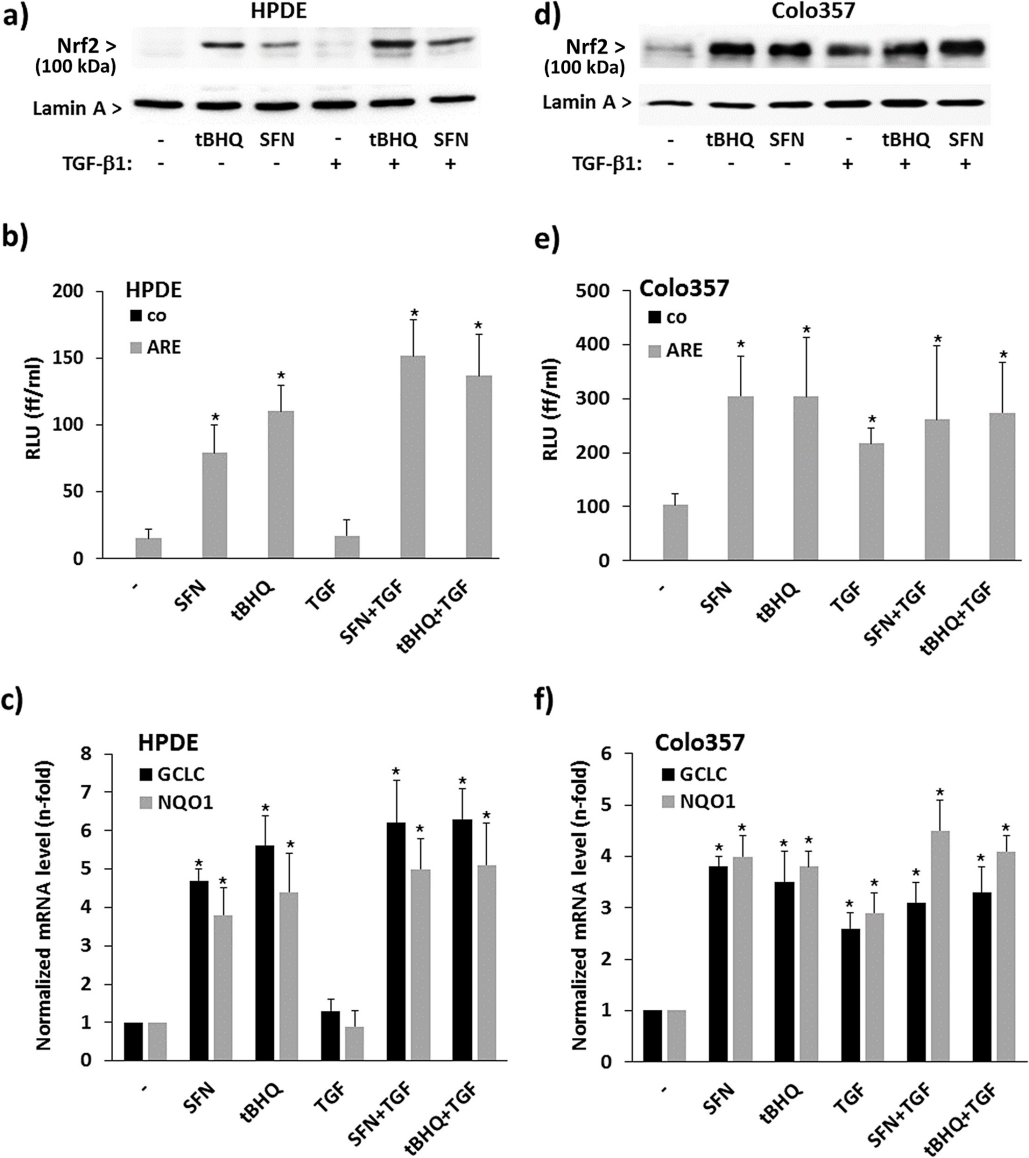
Interference of TGF-β1 with Nrf2 activation in premalignant and malignant pancreatic duct cells. HPDE or Colo357 cells either left untreated or treated for 8h with 50 μM tBHQ, 10 μM SFN or 10 ng/mL TGF-β1, either alone or in combination, were analysed by Nrf2 western blot **(A,D)** using nuclear extracts (lamin-A served as loading control), by ARE-luciferase assay **(B,E)** using an empty vector as control (co) and the pARE vector (ARE), or by real time PCR **(C,F)** for detection of the established Nrf2 target genes NQO1 and GCLC (TBP served as control). In **A)** and **D)** representative results from three independent experiments are shown. A densitometric band intensity evaluation is provided in Figs. A and B in S1 File. In **B)** and **E)** data represent the mean ± SD of four independent experiments performed in duplicate, and in **C)** and **F)** data represent the mean ± SD of six independent experiments. *p<0.05 (treated versus untreated).

To investigate the status of Nrf2 activation and its crosstalk with TGF-β1, the nuclear protein level of Nrf2 was analysed in the nontumorigenic pancreatic ductal epithelial cell line HPDE exhibiting low Nrf2 activity [33] by westernblot. As shown in Fig 1A, the rather low amount of Nrf2 strongly increased in nuclear extracts from HPDE cells after treatment with either of the established Nrf2 inducers tBHQ or SFN, but not after TGF-β1 treatment. Coincubation of tBHQ or SFN with TGF-β1 resulted in an even stronger Nrf2 activation as seen after treatment with tBHQ or sulforaphane (SFN) alone. Likewise, ARE-luciferase assays (Fig 1B) revealed an increase of Nrf2 activity in HPDE cells when treated with tBHQ or SFN but not with TGF-β1, and an even stronger Nrf2 activity after combined treatment. In accordance with the increased Nrf2 activity, expression of established Nrf2 target genes such as NQO1 and GCLC was upregulated in HPDE cells by tBHQ or SFN (Fig 1C) and even more by the combination of Nrf2 inducer and TGF-β1, but not by TGF-β1 alone.

Compared to HPDE cells, the nuclear level of Nrf2 was already much greater in Colo357 cells and further elevated by treatment with tBHQ or SFN (Fig 1D). In contrast to HPDE cells, greater Nrf2 activation was also seen with TGF-β1 whereas no further increase was oberserved after combined treatment with tBHQ and TGF-β1 or SFN. ARE-Luciferase revealed an increase in Nrf2 activity after treatment with either tBHQ, SFN or TGF-β1 (Fig 1E), but no further increase by combined treatments. NQO1 and GCLC expression was induced by tBHQ, SFN or TGF-β1 to similar extent, and no enhancing effect was seen by combining Nrf2 inducers and TGF-β1 (Fig 1F).

### Nrf2 activation and TGF-β1 alter morphology and migration of premalignant pancreatic duct (HPDE) cells in an additive fashion

**Fig 2:**
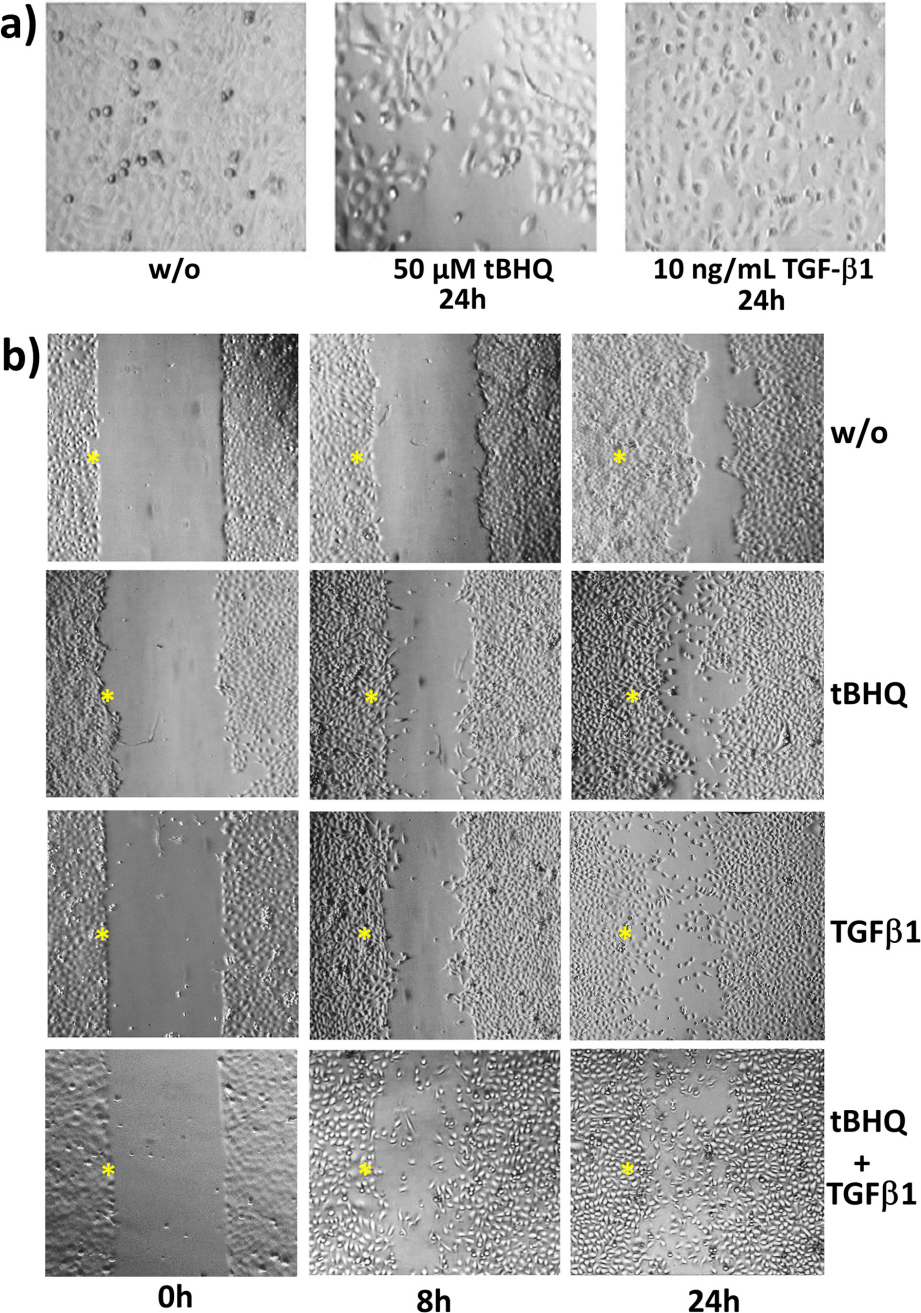
HPDE cell morphology and wound healing after Nrf2 activation by tBHQ or TGF-β1 treatment. **A)** HPDE cells were treated with 50 μM tBHQ or 10 ng/mL TGF-β1, or were left untreated for 24h. Then, cells were analysed by microscopy (at 200x magnification) and photographs were taken. **B)** Confluently grown HPDE cells in a two-chamber insert were treated with 50 μM tBHQ or 10 ng/mL TGF-β1, either alone or in combination, or were left untreated. Then, the insert was removed (t = 0h) and selected areas were analysed by microscopy (at 100x magnification) and photographed at the indicated periods. *marks the intitial wound edges.

In line with the different Nrf2 expression and activity, Colo357 cells are characterized by higher migratory and invasive properties compared to HPDE cells [52]. Thus, to elucidate whether Nrf2 activation leads to alterations in cell morphology and migration of premalignant cells, too, and how this compares with TGF-β1, HPDE cells were incubated with the most strongest Nrf2 activator tBHQ or with TGF-β1 for several periods. Within 24h, tBHQ and TGF-β1 treated cells exhibited similar morphological alterations characterized by loss of cell-cell contacts and a spindle shaped morphology (Fig 2A).

Next, confluent HPDE cells were subjected to wound-healing assay and treatment with tBHQ or TGF-β1 (Fig 2B). Already 8 hours after wounding, untreated HPDE cells began to close the wound by forming a smooth and quite uniform fringe without any signs of single cells invading into the gap, a process which was almost finished after 24 hours. In contrast, tBHQ treated cells started to close the gap much faster and most importantly single cells emerging within the wound. After 24 hours, tBHQ treated HPDE cells migrated frequently into the wound leading to an almost complete gap closure (Fig 2B). Similar effects on the wound healing were noted with HPDE cells treated with TGF-β1 (Fig 2B). The most prominent effect was observed with HPDE cells subject to tBHQ and TGF-β1 co-stimulation (Fig 2B). Compared to the single treatments, the highest amount of single elongated HPDE cells within the wound could be observed after combined treatment already after 8 hours leading to gap closure after 24 hours. Moreover, in contrast to dense monolayer of untreated HPDE cells, the cell layer of tBHQ and TGF-β1 co-stimulated HPDE cells was less dense and showed larger intercellular spaces.

### Effect of Nrf2 and TGF-β1 on the invasiveness of premalignant and malignant pancreatic duct cells

**Fig 3:**
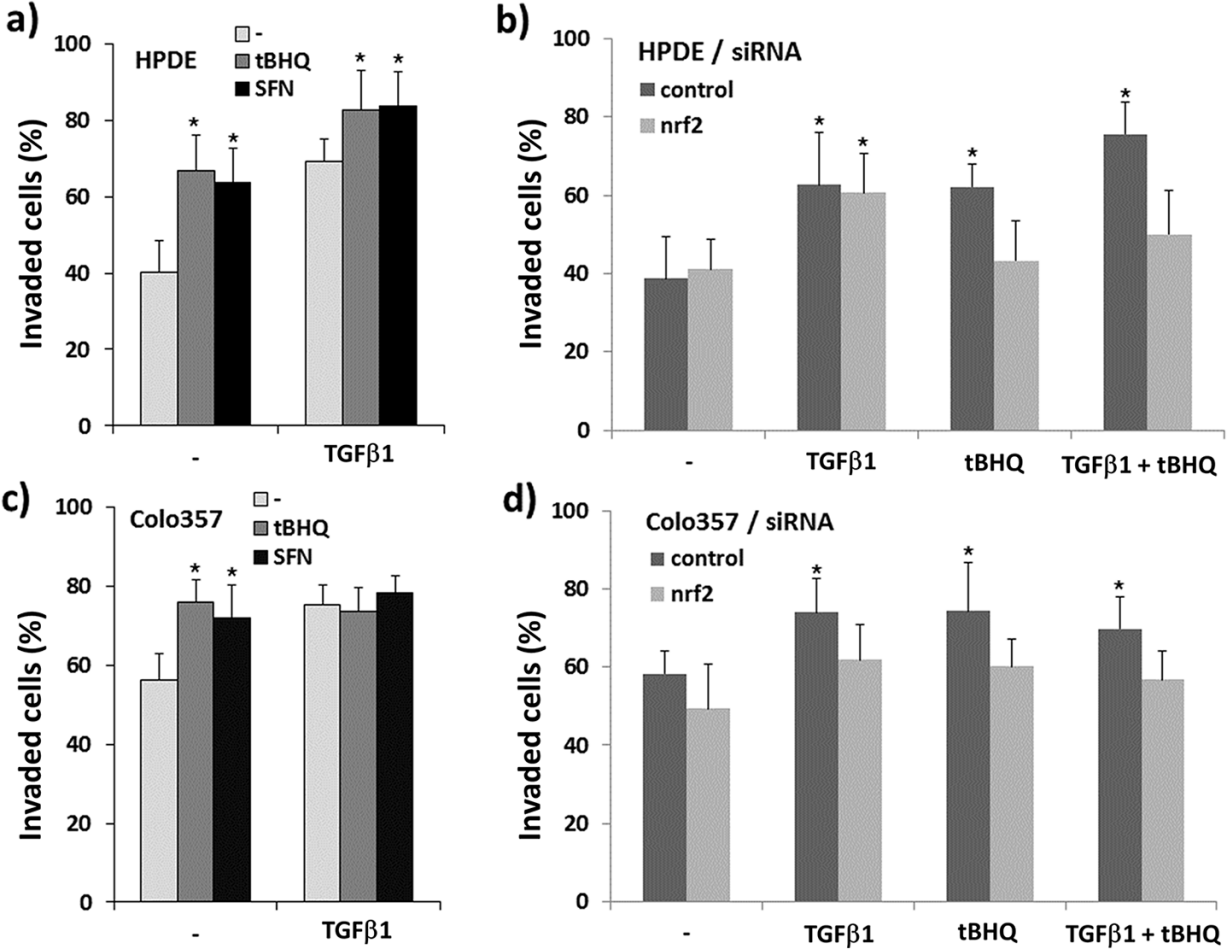
Effect of Nrf2 activation and TGF-β1 on the invasiveness of premalignant HPDE and malignant Colo357 pancreatic duct cells. Modified Boyden chamber assays on collagen-I coated transwells were performed with **A)** HPDE or **C)** Colo357 cells treated either alone or in combination with 50 μM tBHQ, 10 μM SFN or 10 ng/mL TGF-β1, or left untreated for 24h. Data are expressed as percentage of invaded cells and represent the mean ± SD from eight independent experiments, *p<0.05 (+tBHQ and +SFN versus–tBHQ and–SFN, respectively). Boyden assays were also conducted with **B)** HPDE cells or **D)** Colo357 cells subject to Nrf2 or control siRNA treatment for 48h (westernblot verification shown in Figs. A and B in S2 File) prior to further treatments with 50 μM tBHQ and/or 10 ng/mL TGF-β1. Data are expressed as percentage of invaded cells and represent the mean ± SD from six independent experiments, *p<0.05 (treated versus untreated).

To substantiate the findings on cell migration, cell invasion through a collagen-I matrix was analysed by a Boyden chamber assay. Both tBHQ and TGF-β1 treatment significantly increased the invasiveness of HPDE cells from 40.2 ± 8.4% to 66.7 ± 9.3% and 69.2 ± 5.8%, respectively. After combined treatment with tBHQ and TGF-β1, the number of invaded cells further increased to 82.7 ± 10.3% (Fig 3A). To a similar extent, SFN as another Nrf2 activator increased the invasion rate of HPDE cells to 63.7 ± 8.8% when given alone and to 83.4 ± 9.1% when added together with TGF-β1.

To verify the Nrf2 dependency of these effects and to exclude side effects by tBHQ as the more potent inducer of invasion, Boyden assays were performed with HPDE cells pre-treated with Nrf2 siRNA (Fig 3B & Fig A in S2 File). Compared to control siRNA cells exhibiting an increase of the invasion rate from 38.7 ± 10.1% to 62.2 ± 5.9% when treated with tBHQ, Nrf2 siRNA cells showed no such difference after tBHQ administration (41.0 ± 7.6% versus 43.3 ± 10.2%). No diminishing effect by the Nrf2 siRNA pre-treatment was seen with basal or TGF-β1 induced invasion that was nearly unaffected (62.5 ± 13.5% versus 60.6 ± 10.0%). However, Nrf2 siRNA potently decreased the enhanced invasion seen after the combined tBHQ and TGF-β1 treatment (50.2 ± 11.7% compared to 75.6 ± 7.9%). Thus, Nrf2 is required for the tBHQ but not the TGF-β1 induced invasion of HPDE cells that independently, but additively, induce this phenotype.

To explore how tBHQ and TGF-β1 alone or in combination influence the invasion of malignant pancreatic duct cells, too, we made use of Colo357 cells which is characterized by an already amplified Nrf2 activity [33,34]. As shown by Boyden assay, the invasion rate of Colo357 cells increased from 56.3 ± 6.7% to 75.8 ± 5.6%, 72.1 ±8.3% and 75.2 ± 5.6% when treated with tBHQ, SFN and TGF-β1, respectively (Fig 3C). Combined treatment of tBHQ or SFN with TGF-β1 did not result in a further increase of Colo357 cell invasion (73.6 ± 6.0% or 77.8 ± 4.4%).

Upon pre-treatment with Nrf2 siRNA (Fig 3D & Fig B in S2 File), already the basal invasion rate of Colo357 cells decreased compared with control siRNA pre-treatment (49.3 ± 11.4% versus 58.4 ± 9.1%). Likewise, the inducing effect of tBHQ on the invasion of Colo357 cells was decreased (60.1 ± 7.0% versus 74.3 ± 12.6%). Intriguingly, the effect of TGF-β1 alone was also reduced by Nrf2 siRNA as compared to control siRNA (61.7 ± 9.1 versus 74.1 ± 8.6%). The combined treatment with tBHQ and TGF-β1 was ineffective, too, when Colo357 cells had been subject to Nrf2 siRNA pre-treatment (56.7 ± 7.4% versus 69.8 ± 8.4%). Thus, in Colo357 cells, Nrf2 activation and TGF-β1 induced an invasive phenotype in a synergistic fashion.

### Interference of Nrf2 with basal and TGF-β1 dependent EMT marker expression in premalignant and malignant pancreatic duct cells

**Fig 4:**
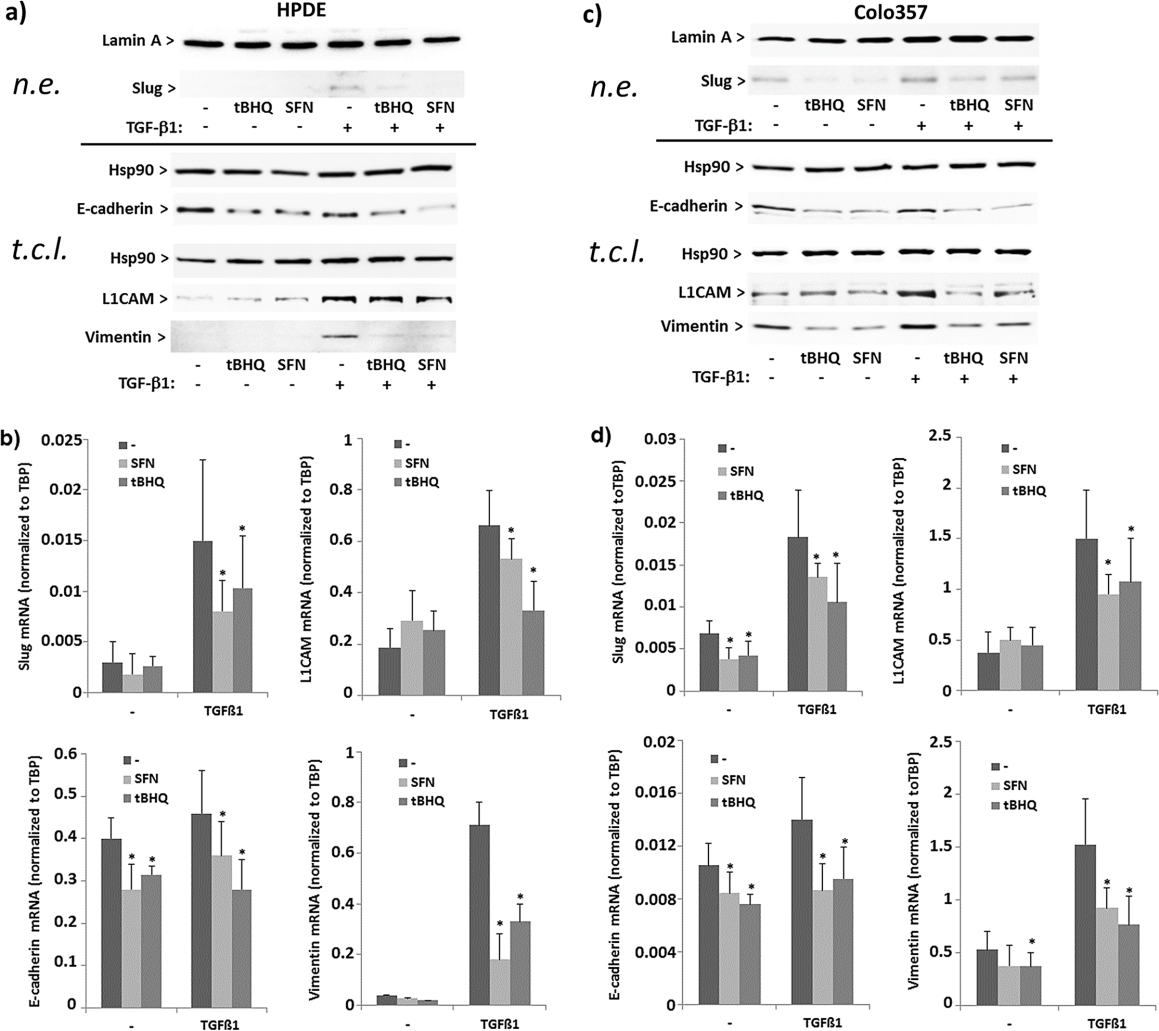
Effect of Nrf2 activation and TGF-β1 on the expression of EMT markers in premalignant HPDE and malignant Colo357 pancreatic duct cells. HPDE or Colo357 cells incubated with 50 μM tBHQ, 10 μM SFN or 10 ng/mL TGF-β1 alone or in combination or without for 24h. Then, either nuclear extracts (n.e.) or total cell lysates (t.c.l.) were analysed by westernblot (**A,C**), or RNA samples were analysed by real-time PCR (**B,D**) for the expression of Slug, L1CAM, E-cadherin or vimentin. Lamin-A/C and Hsp90 were used as loading controls for the westernblots of nuclear extracts and total cell lysates, respectively (**A,C**), and for normalization of Slug, L1CAM, E-cadherin and vimentin mRNA level TBP was analysed in parallel (**B,D**). Either a representative result from three independent experiments (**A,C**) or the mean ± SD from six independent experiments (**B,D**) are shown, *p<0.05 (+ tBHQ and + SFN versus–tBHQ and–SFN, respectively); (**A,C**) a densitometric band intensity evaluation is provided in Figs. A and B in S3 File.

Next, it was investigated whether Nrf2 and TGF-β1 mediated effects on cell invasion relate to an altered expression of EMT marker (vimentin, L1CAM, Slug, E-cadherin) expression in HPDE cells. Westernblot and qPCR analysis revealed that after treatment with tBHQ (50 μM) or SFN (10 μM) the expression of vimentin and Slug remained at low basal level, whereas the expression of E-cadherin decreased and the expression of L1CAM slightly increased (Fig 4A and 4B). By contrast, treatment of HPDE cells with TGF-β1 (10 ng/mL) resulted in an increase of vimentin and Slug expression, as well as of L1CAM. No marked effect was seen on E-cadherin expression after TGF-β1 treatment. After combined treatment with TGF-β1 and tBHQ or SFN, the increase of vimentin and Slug expression was lower compared to the effect by TGF-β1 treatment alone. The E-cadherin expression was even more strongly decreased by the combined treatments than by tBHQ or SFN treatment alone. L1CAM expression was slightly lower after combined treatment with TGF-β1 and tBHQ or SFN compared to treatment with TGF-β1 alone (Fig 4A and 4B).

In Colo357 cells, basal vimentin and Slug expression were significantly reduced by tBHQ and SFN, whereas TGF-β1 further increased the expression of these two EMT markers. In the presence of tBHQ or SFN the inducing effect of TGF-β1 on vimentin and Slug expression was diminished. Likewise, the expression of L1CAM was greater in Colo357 than in HPDE cells and further increased after TGF-β1 treatment. The administration of the Nrf2 activators only marginally affected basal L1CAM expression but significantly reduced the TGF-β1 induced L1CAM expression. E-cadherin expression being per se lower in Colo357 cells than in HPDE cells further declined after administration of tBHQ or SFN, did not change significantly after TGF-β1 treatment and was strongly downregulated after combined treatment with tBHQ and TGF-β1. (Fig 4C and 4D).

**Fig 5:**
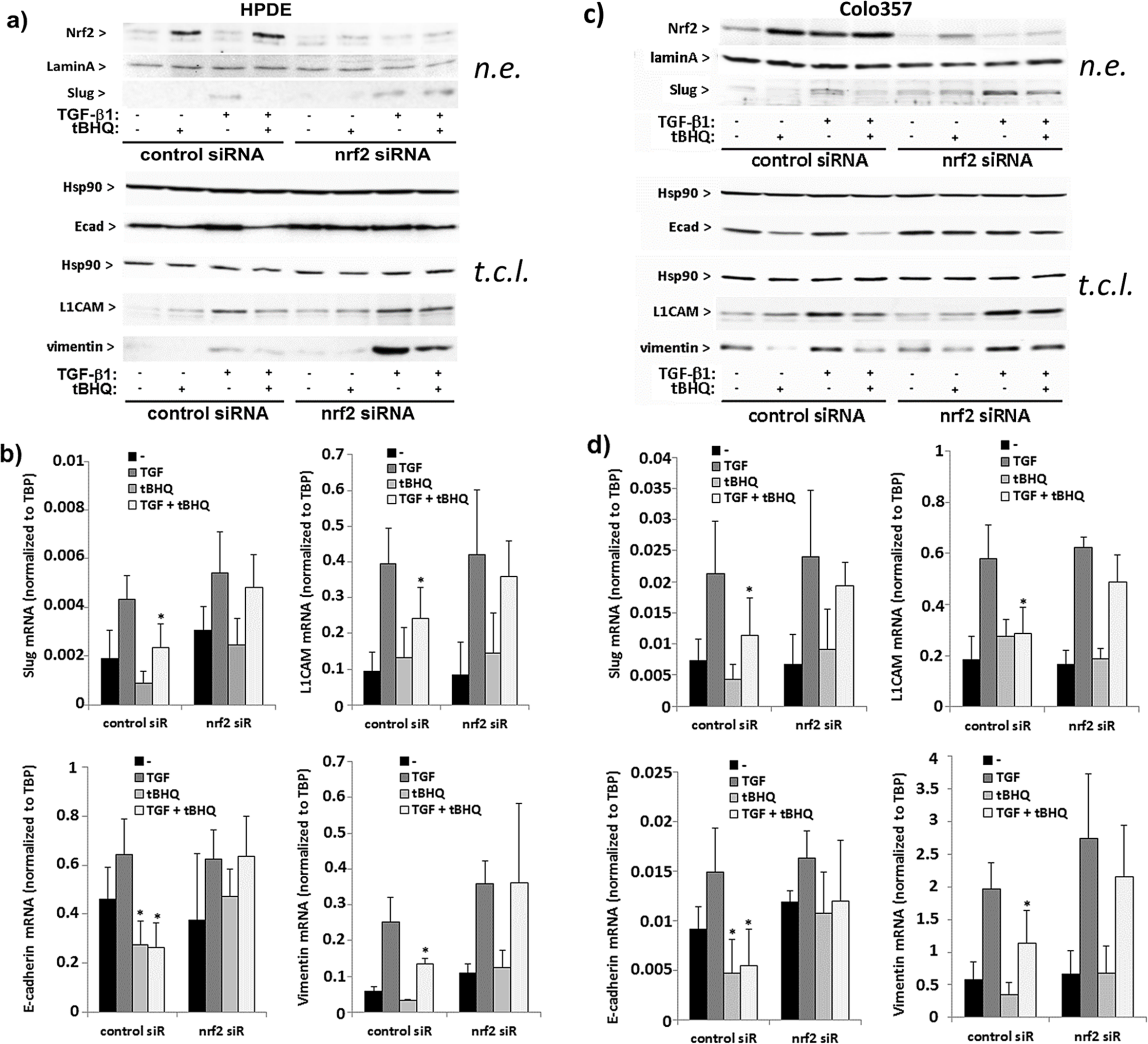
Nrf2 dependency of the tBHQ effects on EMT marker expression in premalignant HPDE and malignant Colo357 pancreatic duct cells. HPDE or Colo357 cells incubated with Nrf2 or control siRNA for 48h were further treated for 24h with tBHQ or TGF-β1, either alone or in combination, or were left untreated. Then, either nuclear extracts (n.e.) or total cell lysates (t.c.l.) were analysed by westernblot (**A,C**), or RNA samples were analysed by real-time PCR (**B,D**) for the expression of Slug, L1CAM, E-cadherin or vimentin. Lamin-A/C and Hsp90 were used as loading controls for the westernblots of nuclear extracts and total cell lysates, respectively (**A,C**), and for normalization of Slug, L1CAM, E-cadherin and vimentin mRNA level TBP was analysed in parallel (**B,D**). Either a representative result from three independent experiments (**A,C**) or the mean ± SD from six independent experiments (**B,D**) are shown, *p<0.05 (+tBHQ versus–tBHQ). (**A,C**) a densitometric band intensity evaluation is provided in Figs. A and B in S4 File.

In the presence of Nrf2 siRNA, the effects of tBHQ on EMT marker expression on protein as well as on mRNA level were diminished in HPDE (Fig 5A and 5B) and Colo357 cells (Fig 5C and 5D) supporting the Nrf2 dependency. The observation that the effect of TGF-β1 on E-cadherin expression was not significantly affected by the Nrf2 knockdown in Colo357 cells may be explained by the fact that the alteration of E-cadherin expression after TGF-β1 treatment occurs more delayed as compared to the effects seen with Nrf2 inducers or as compared with the other EMT markers.

### Interference of Nrf2 with basal and TGF-β1 dependent Smad and JNK signalling pathways in premalignant and malignant pancreatic duct cells

**Fig 6:**
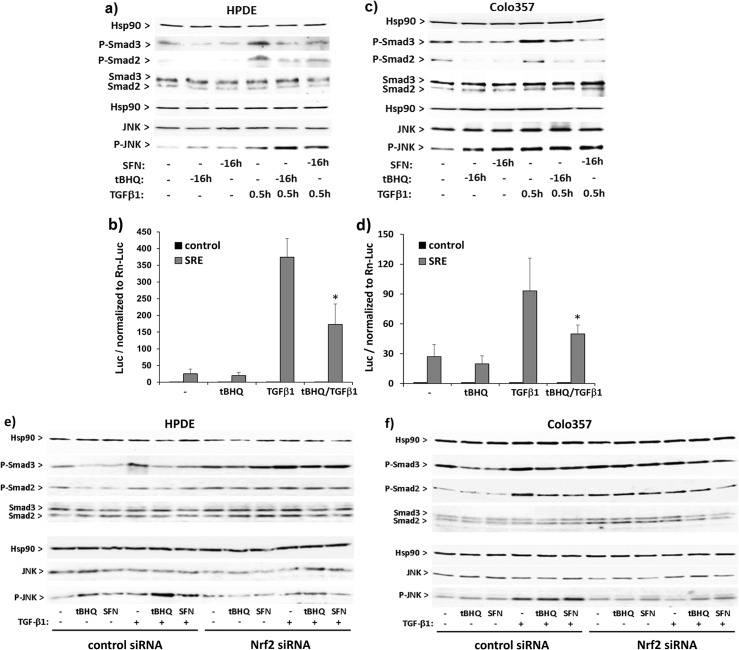
Nrf2 activation affects basal and TGF-β1 dependent Smad and JNK signalling pathways in premalignant and malignant pancreatic duct cells. **A)** HPDE or **B)** Colo357 cells incubated with 50 μM tBHQ, 10 μM SFN or 10 ng/mL TGF-β1 alone or in combination for the indicated periods. Then total cell lysates were analysed by westernblot (**A,C**) for expression of P-Smad2, P-Smad3, Smad2/3, P-JNK and JNK using Hsp90 as loading control. Representative results from three independent experiments are shown and a densitometric band intensity evaluation is provided in Figs. A and B in S5 File. **B)** HPDE or **D)** Colo357 cells were transfected with pGL3 (control) or p6SBE (SRE) together with ptkRL followed by incubation with 50 μM tBHQ and/or 10 ng/mL TGF-β1 or without. After 16h, cell lysates were analysed for firefly and renilla luciferase expression and firefly luciferase units were normalized to those of renilla luciferase. Data represent the mean ± SD of six independent experiments performed in duplicate, p<0.05 (+tBHQ versus-tBHQ). **E)** HPDE or **F)** Colo357 cells were treated with Nrf2 or control siRNA for 48h, followed by incubation with 50 μM tBHQ, 10 μM SFN and/or 10 ng/mL TGF-β1, or without. Then, total cell lysates were analysed by westernblot for expression of P-Smad2, P-Smad3, Smad2/3, P-JNK and JNK using Hsp90 as loading control. Representative results from three independent experiments are shown, and a densitometric band intensity evaluation is provided in Figs. C and D in S5 File.

To elucidate whether the interference of tBHQ or SFN treatment with EMT marker expression in response to TGF-β1 relates to an altered Smad or MAPK signalling, westernblot analyses were conducted. As shown in Fig 6A, tBHQ or SFN treatment (16 h) decreased basal phospho-Smad3/2 level in HPDE cells and blocked the increasing effect by TGF-β1 treatment (0.5 h). Smad response element (SRE)-luciferase assays confirmed the negative interference of Nrf2 activation with the Smad-pathway in HPDE cells (Fig 6B) as shown by the attenuating effect of tBHQ treatment on TGF-β1 induced SRE-driven luciferase expression.

In turn, activation of JNK, representing an alternative signalling pathway of TGF-β1, was moderately induced by TGF-β1 as well as by tBHQ or SFN, as shown by the slight increase of phospho-JNK protein level (Fig 6A). The combined treatment of HPDE cells with TGF-β1 and tBHQ or SFN strongly enhanced the upregulation of phospho-JNK levels seen with these stimuli alone (Fig 6A).

In Colo357 cells, tBHQ or SFN similarly inhibited Smad signalling as shown by phospho-Smad3/2 westernblot and SRE-luciferase assay (Fig 6C and 6D). In contrast to HPDE cells, JNK phosphorylation was augmented to similar extent by the Nrf2 activators or by TGF-β1 either given alone or in combination, thus indicating greater contribution of Smad independent signalling to the effects of TGF-β1 in this PDAC cell line.

When knocking down Nrf2 expression by Nrf2 siRNA treatment, the decreasing effects of tBHQ or SFN on the protein level of phospho-Smad2/3 in both HPDE and Colo357 cells (Fig 6E and 6F, respectively) were markedly reduced as compared to cells treated with control siRNA. Interestingly, the enhancing effects of tBHQ or SFN on JNK activation by TGF-β1 in HPDE cells as well as the TGF-β1 induced JNK-phosphorylation in Colo357 cells were abrogated after Nrf2 knock down (Fig 6E and 6F, respectively).

### The human E-cadherin promoter contains an inhibitory Nrf2 binding site (ARE)

**Fig 7:**
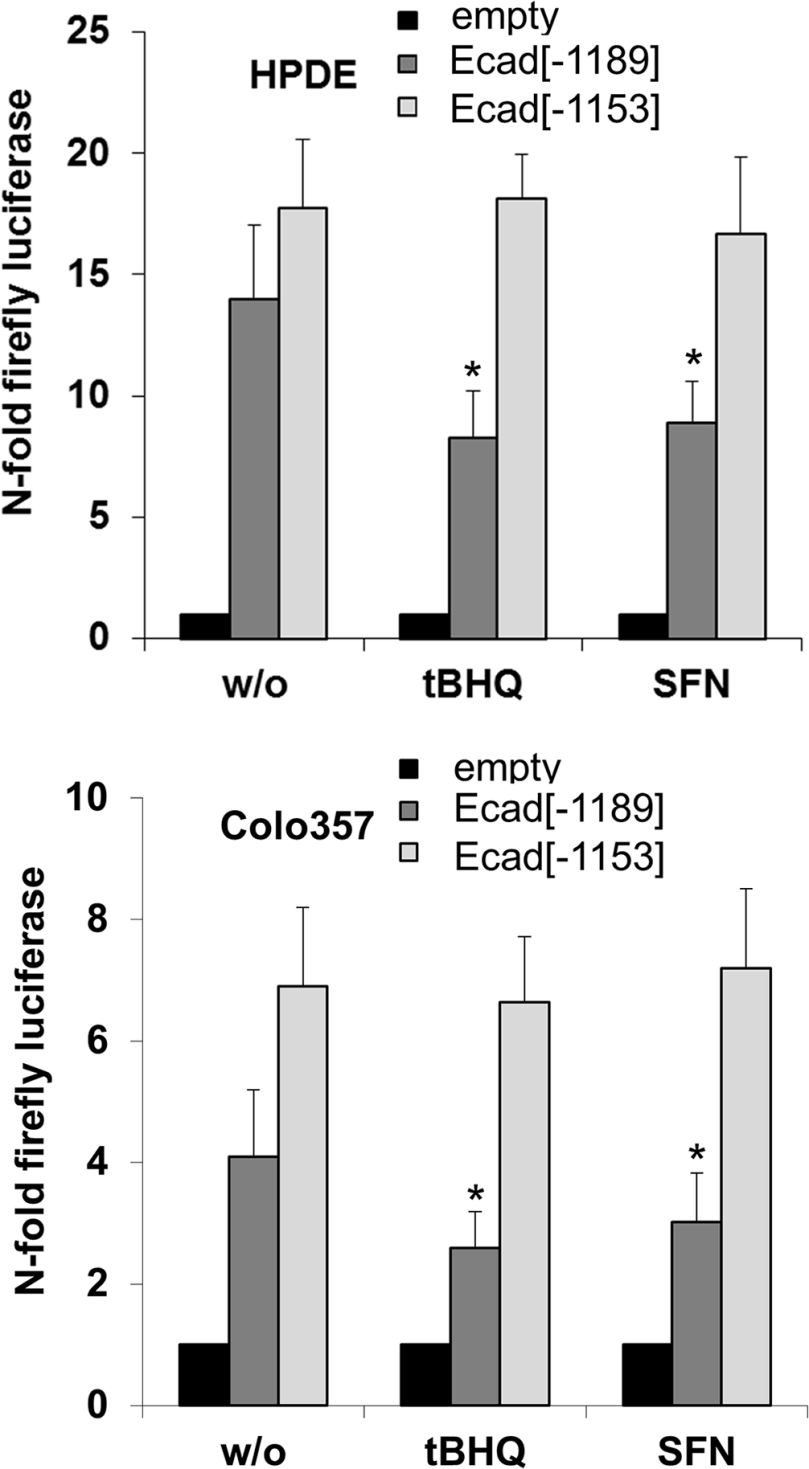
A Nrf2 binding site in the human E-cadherin promoter exerts transcriptional repression. HPDE **(A)** or Colo357 **(B)** cells were transfected with firefly luciferase reporter gene constructs containing the ARE like site (Ecad[–1189]) from the E-cadherin promoter, or not (Ecad[–1153]), or with the empty reporter gene vector. Cells were left untreated or were treated with tBHQ or SFN for 8h. Then, firefly luciferase activity was measured and normalized to renilla luciferase. Data represent the mean of 4 independent experiments. A scheme of the cloned E-cadherin promoter fragments is provided in Figs. A and B in S6 File.

Since the Nrf2 mediated downregulation of E-cadherin expression did not correlate with the effects of Nrf2 on Smad and JNK signalling, we next elucidated whether Nrf2 directly impacts on E-cadherin expression by analysing the gene promoter of human E-cadherin for potential Nrf2 binding sites (ARE). We could identify an ARE related motif at position -1153 to -1162 (Fig A in S6 File). To study the impact of this site on transcriptional activity, luciferase assays were conducted using constructs either containing (Ecad[–1189]) or lacking this site (Ecad[–1153]). As shown in Fig 7, the luciferase expression in HPDE cells transfected with Ecad[–1189] was slightly lower than in HPDE cells transfected with Ecad[–1153]. Treatment with tBHQ or SFN further reduced the luciferase activity in Ecad[–1189] transfected but not in Ecad[–1153] transfected HPDE cells. Already in the absence of tBHQ or SFN, luciferase expression was significantly lower in Colo357 cells transfected with Ecad[–1189] as compared to Ecad[–1153] transfected Colo357 cells. Again, tBHQ or SFN treatment further decreased luciferase expression in Colo357 cells transfected with Ecad[–1189] but not in Colo357 cells transfected with Ecad[–1153].

These findings point to a direct interference of Nrf2 with the activity of the E-cadherin promoter through an ARE site.

### The Nrf2 dependent effect on the invasiveness relates to the deregulation of E-cadherin

**Fig 8:**
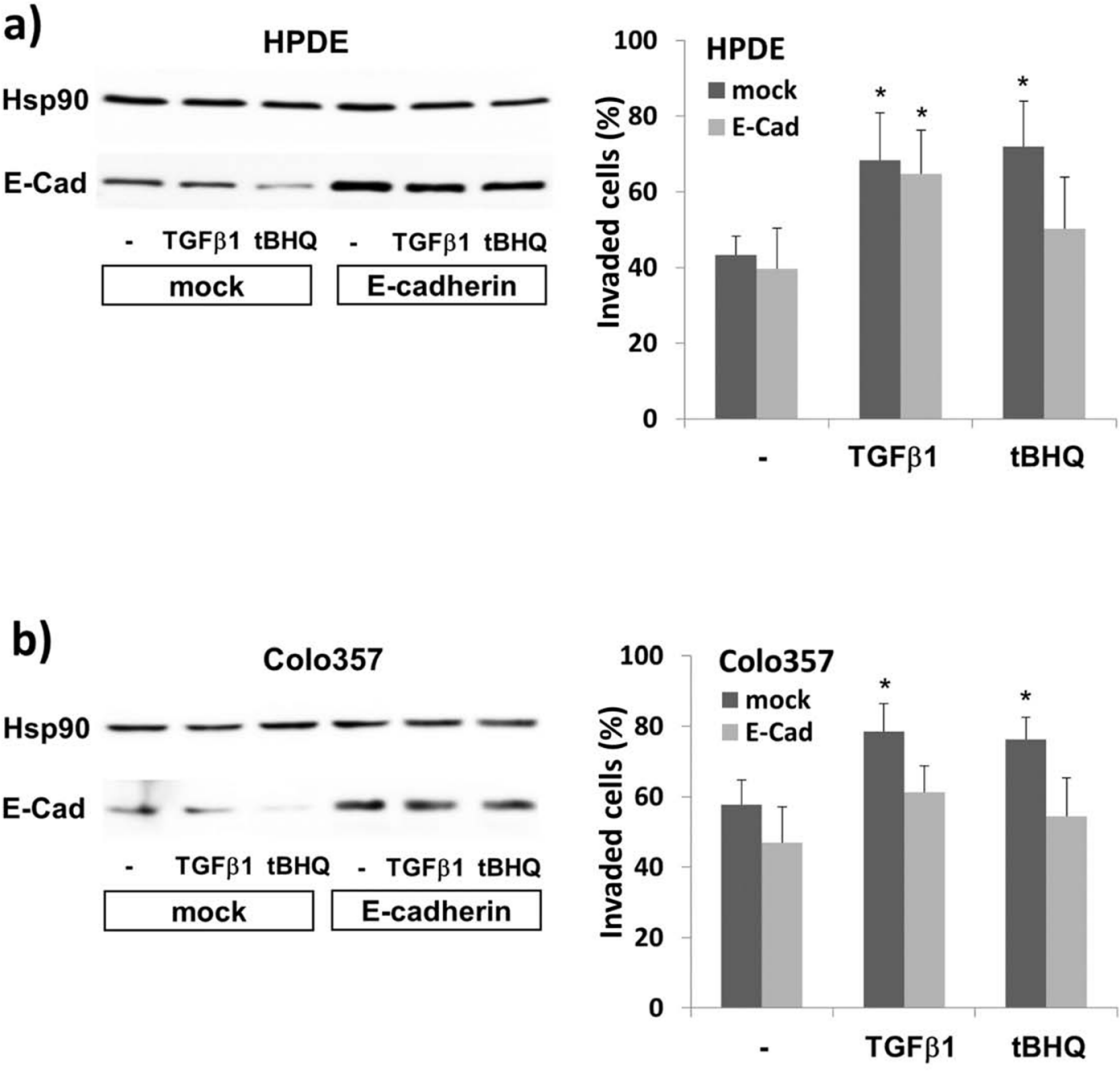
Maintained expression of E-cadherin affects the inducing effect of Nrf2 activation on the invasion of premalignant and malignant pancreatic duct cells. HPDE **(A)** or Colo357 **(B)** cells were transfected with a constitutive E-cadherin expression vector or an empty pcDNA3.1 vector (mock). Afterwards, cells were incubated with 50 μM tBHQ or 10 ng/mL TGF-β1 for 24h, or were left untreated. Then, cells were submitted to the modified Boyden assay on collagen-I coated transwells (**A,B**, right panels). In parallel, total cell lysates were analysed by E-cadherin western blots (**A,B**, left panels) using Hsp90 as loading control. A densitometric band intensity evaluation is provided in Figs. A and B in S7 File. The westernblots show representative results from four independent experiments. The invasion data (**A,B**, right panels) represent the mean ± SD of four independent experiments performed in duplicate, *p<0.05 (treated versus untreated).

The observation that Nrf2 activation directly decreases the expression of E-cadherin but does not upregulate mesenchymal markers like vimentin prompted us to look whether the proinvasive effect of Nrf2 depends on the decline of E-cadherin expression. For this purpose, HPDE and Colo357 cells were transfected with E-cadherin to ensure its sustained expression during treatment with tBHQ or TGF-β1 for 24h. Boyden chamber analyses (Fig 8A) revealed that the inducing effect of tBHQ on HPDE cell invasion was significantly decreased by E-cadherin overexpression. In mock-transfected HPDE cells the basal invasion rate (42.3 ± 5.3%) increased 1.67 fold after tBHQ treatment (72.2 ± 12.1%) whereas in E-cadherin transfected HPDE cells the invasion rate increased only 1.25-fold from 39.7 ± 10.2% to 50.1 ± 13.7%. Interestingly, TGF-β1 induced migration was only slightly affected by E-cadherin re-expression (64.7 ± 11.5% versus 68.4 ± 12.4%).

In Colo357 cells (Fig 8B), the tBHQ induced invasion rate (mock: 76.2 ± 4.7% compared to 57.7 ± 7.1%) was also reduced by E-cadherin overexpression (54.3 ± 10.8% compared to 47.0 ± 10.1%). Unlike HPDE cells, TGF-β1 treated Colo357 cells revealed a decrease in their invasion rate when E-cadherin was overexpressed (78.5 ± 7.9% versus 61.2 ± 7.5%).

### Reciprocal expression level of activated Nrf2 and E-cadherin in pancreatic duct cells of PanINs in tissues from chronic pancreatitis (CP) patients

To elucidate whether an increased Nrf2 activity in the pancreatic ductal epithelium associates with the decline in E-cadherin expression already at a premalignant stage, e.g. during CP, immunostainings of formalin fixed and paraffin embedded pancreatic tissues from twenty CP patients were conducted. We used an antibody against Phospho-Ser40-Nrf2 that specifically detects activated Nrf2 residing in the nucleus mainly in its Ser40-phosphorylated form, as shown recently for Colo357 tumors [33].

**Fig 9:**
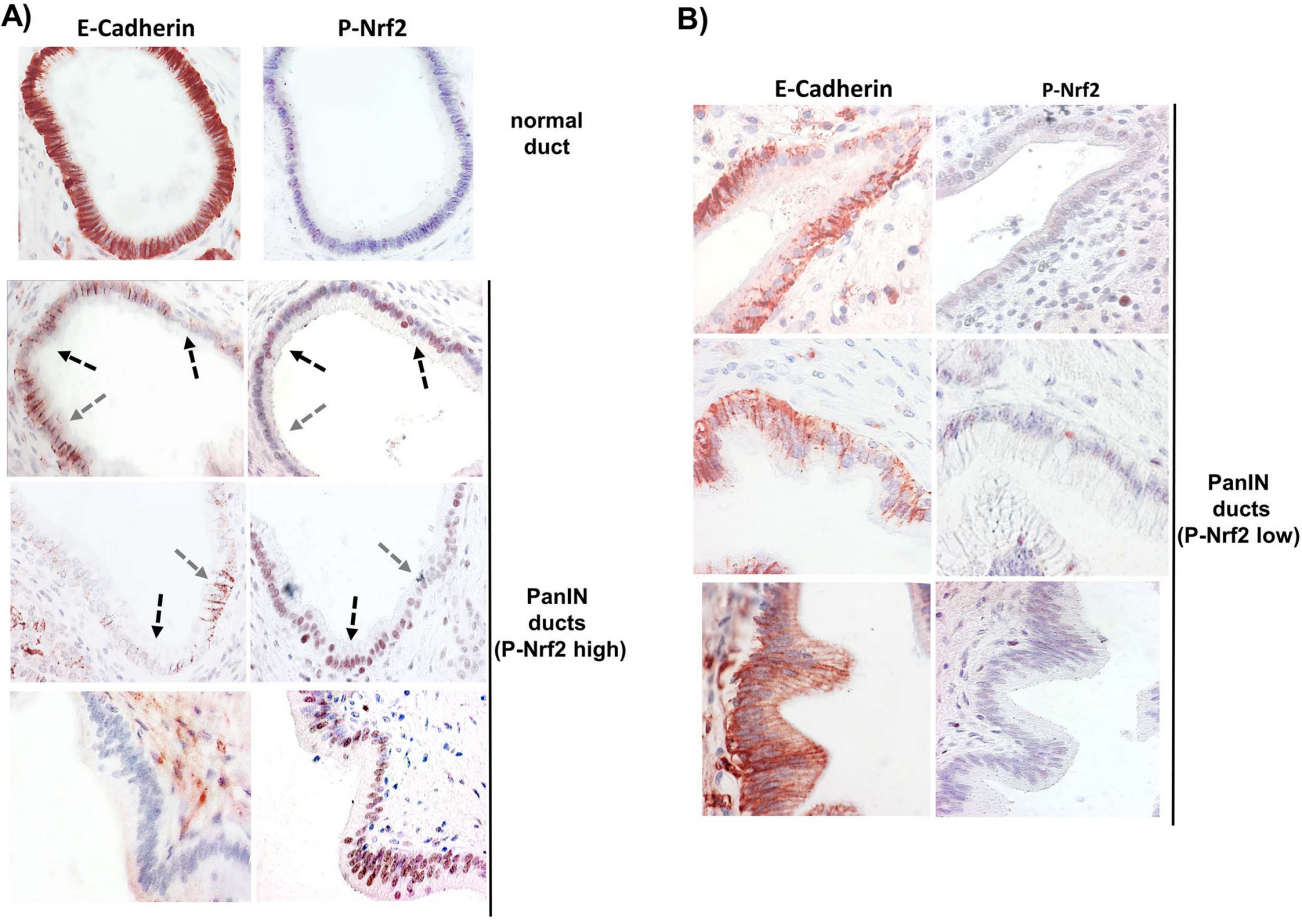
Immunohistochemistry analysis for activated Nrf2 and E-cadherin expression in premalignant pancreatic tissues. Formalin fixed and paraffin embedded pancreatic tissues from CP patients was subjected to immunostaining for P-Nrf2 and E-cadherin. Representative images are shown of **(A)** normal ducts and PanIN lesions exhibiting high expression of P-Nrf2 or **(B)** PanIN lesions with low expression of P-Nrf2 that display the respective reciprocal expression level of E-cadherin. Usage of the isotype matched control antibodies revealed no or only weak background staining (not shown). Images were taken at 400x magnification. Arrows indicate ductal regions of reciprocal P-Nrf2 and E-cadherin expression within the same lesion.

Hereby it was shown that nuclear P-Nrf2 levels were significantly elevated already in the epithelium of PanIN lesions when compared to healthy ducts (Fig 9A). Overall 36 PanIN structures found in 11/20 CP patients were analysed, 23 of which (64%) exhibited considerable P-Nrf2 staining (score >1). E-cadherin expression in these 23 lesions was still high (score >1) only in 9 cases and diminished (score <1) in 14 cases. In those 6 ductal PanIN structures with greatest expression level of nuclear P-Nrf2 (score = 2), E-cadherin expression was almost lost (score = 0). In turn, the 13 PanIN lesions with marginal or undetectable P-Nrf2 activity (score <1) exhibited high (score >1) and low (score <1) E-cadherin expression in 10 and 3 cases, respectively (Fig 9B).

These reciprocal P-Nrf2 / E-cadherin expression patterns in early PanIN lesions indicate that the process of Nrf2 activation and EMT already commences at a premalignant stage and an inflammatory context.

## Discussion

One condition during inflammation associated carcinogenesis is the long term exposure of epithelial cells to an inflammatory microenvironment and their stress adaptation. This adaptation involves cellular mediators that counterregulate the local inflammatory response and protect from cell damage. Amongst these mediators, TGF-β1 and Nrf2 have an important role by primarily acting immunosuppressive and cytoprotective against oxidative stress, respectively. Besides these compensatory effects, TGF-β1 and Nrf2 also confer growth advantages to permanently stressed cells that may favour tumor development and progression. In particular, deregulated activation of Nrf2 that takes place during long-term stress exposure and together with the initiation of oncogenic pathways like kRas or PI3K/Akt as well as the shift of TGF-β1 signalling from a growth suppressive towards a promigratory and proinvasive activity contribute both to tumorigenesis. These protumorigenic effects may occur independently but also in a cooperative fashion.

PDAC represents a tumor entity characterized by an extended stromal compartment that already forms during earliest steps of its development on the basis of a CP and/or PanINs [53,54]. Enriched of diverse immune cells such as macrophages and certain T-cell subsets as well as of myofibroblasts [55–57], the stroma in inflammation associated carcinogenesis of the pancreas essentially affects the phenotype of the ductal epithelium [35]. TGF-β1 is known to initiate tumorigenesis quite early by conferring an invasive phenotype to the epithelial cells which is related to the EMT process. Likewise, Nrf2 confers several growth advantages to premalignant cells presumably in an early state, as well [33,34,58]. In example, HPDE and PDAC cells gain robust protection from apoptosis stimuli after Nrf2 activation, e.g by tBHQ [33], whereas knock down of Nrf2 renders these cells more sensitive to apoptosis.

Addressing the question whether Nrf2 and TGF-β1 act together in malignant transformation we investigated the phenotype and behaviour of the pancreatic duct cell line HPDE and the PDAC cell line that both exhibit TGF-β1 responsiveness unaffected by deletion or mutation of SMAD4 [59]. We observed a similar effect of Nrf2 induction and TGF-β1 treatment on HPDE cells with respect to cell morphology, migration and invasiveness. The observed effects were independent of each other but work in an additive fashion. Thus, already in premalignant cells Nrf2 is capable of promoting a malignant phenotype that is further enhanced by TGF-β1. In Colo357 cells, Nrf2 activity is already deregulated and obviously shapes the TGF-β1 responsiveness of this PDAC cell line. This relates to the inhibitory effect of Nrf2 on the canonical Smad signalling pathway [40,41] that also abrogates the anti-proliferative effect of TGF-β1 (H. Schäfer & S. Sebens, unpublished observation). Conversely, Nrf2 amplifies the inducing effect of TGF-β1 on the non-canonical pathway, as indicated by the enhanced activation of JNK, thereby favouring a motile and invasive phenotype in response to TGF-β1 treatment. This proinvasive effect of JNK activation by TGF-β1 on HPDE and Colo357 cells was shown previously [35]. The mechanisms by which Nrf2 switches TGF-β1 signalling that way are still unknown and deserve further investigation.

The observed impact of Nrf2 on the proinvasive effect of TGF-β1 and JNK activation represents a novel mechanism by which TGF-β1 and Nrf2 may synergize in malignant transformation. Interestingly, this synergism between Nrf2 and TGF-β1 in Colo357 cells and also their additive effect in HPDE cells is dispensable of some classical EMT associated alterations such as greater expression of Slug and vimentin seen with TGF-β1 treatment alone.

Instead, downregulation of E-cadherin is efficiently promoted by Nrf2 activation that obviously dominates the suppressive effects of Nrf2 on Slug and vimentin expression. Moreover, unlike glioma cells [44], HPDE and Colo357 cells revealed no inducing effect on MMP expression by Nrf2 underlying its promigratory effect. Thus, it was tempting to speculate that the desintegration of the cell-cell interactions by the Nrf2 dependent decrease of E-cadherin expression is sufficient to confer a motile and invasive phenotype to HPDE and Colo357 cells. Indeed, overexpression of E-cadherin in HPDE and Colo357 cells suppressed the proinvasive effects by Nrf2 activation. When combining Nrf2 activation and TGF-β1 treatment, TGF-β1 adds its proinvasive effect in HPDE cells probably through a residual EMT inducing activity, whereas in Colo357 cells Nrf2 is the prevailing determinant of the interdependent effects of Nrf2 and TGF-β1 on the motile phenotype. Obviously, the anti-migratory and EMT inhibitory effects of Nrf2, as reported by contrast in hepatoma cells and myofibroblasts [40,41], rely on the blockade of Smad-dependent signalling, but under conditions of E-cadherin suppression, as in Colo357 and HPDE cells, Nrf2 may favour a migratory phenotype.

The finding that the expression of E-cadherin is decreased by Nrf2 activation already on the mRNA level indicated that Nrf2 either directly affects activation of the E-cadherin promoter or that Nrf2 exerts epigenetic effects, e.g. through miRs like miR-10b or miR-208. Intriguingly, we identified an ARE/EpRE-like region in the promoter region of the E-cadherin gene (position -1153 to -1162) that seems to be involved in Nrf2 dependent modulation of E-cadherin expression. In the presence, but not in the absence, of this site, E-cadherin promoter activity was greatly reduced by Nrf2 activation (Fig 7). Thus, Nrf2 has a direct suppressive effect on E-cadherin expression. Supporting this idea, Shen et al. recently reported a reciprocal link between Nrf2 and E-cadherin in esophageal cancer cells relating to the pro-invasive potential of Nrf2 [24]. Moreover, our immunohistochemistry analysis revealed already in premalignant PanIN lesions of CP tissues an enhanced Nrf2 activation accompanied by a decreased E-cadherin expression. This indicates that the gain of Nrf2 activity and the concomitant loss of E-cadherin expression—representing common features of advanced PDAC [30,60–62] and indicative for a more aggressive phenotype—occur quite early during the initiation of pancreatic carcinogenesis, e.g. when originating from chronic inflammation. Besides the decreasing E-cadherin expression, vimentin and L1CAM expression slightly increased in these PanINs, as shown previously [63]. Intriguingly, the expression of these three markers was not concomitantly altered in the same PanIN. Thus, mesenchymal proteins were still hardly detectable in these epithelial cells despite an already diminished E-cadherin expression and vice versa. Thus, E-cadherin downregulation and mesenchymal marker upregulation may occur independently, a process in which the downregulation of E-cadherin is directly controlled by Nrf2.

Overall, it is tempting to speculate that the crosstalk between Nrf2 and TGF-β1 is shifted from a mutually inhibiting one towards an additive one–as demonstrated in premalignant HPDE cells, and later on towards a Nrf2 dominated one–as shown for Colo357 cells. This could be a hallmark of inflammation associated carcinogenesis where Nrf2 and TGF-β1 are part of stress and inflammation compensating mechanisms first, but then become essential part of cellular events that initiate and promote tumor formation. Such a connection may also have implications of therapeutic strategies that could make use of recently introduced small molecule inhibitors of Nrf2 [33,64–66], thereby preventing the deleterious “alliance” with TGF-β1.

## Supporting Information

S1 File Densitometric evaluation. Densitometric evaluation (Quantity One software, Bio-Rad) of Nrf2 protein band intensities (normalized to LaminA) from westernblot analyses of nuclear extracts from HPDE cells (**Fig A)** and Colo357 cells (**Fig B)** cells. Mean values of three independent experiments are shown.
(PDF)

S2 File Nrf2 westernblot analysis. Nuclear extracts from HPDE cells (**Fig A)** and Colo357 cells (**Fig B)** treated with Nrf2 or control siRNA were submitted to Nrf2 westernblot; lamin A was used as loading control.
(PDF)

S3 File Densitometric evaluation. Densitometric evaluation (Quantity One software, Bio-Rad) of the indicated protein band intensities from westernblot analyses of nuclear extracts (Slug) or total lysates (L1, E-cadherin, vimentin) from HPDE cells (**Fig A)** and Colo357 cells (**Fig B)**. Mean values of three independent experiments are shown.
(PDF)

S4 File Densitometric evaluation. Densitometric evaluation (Quantity One software, Bio-Rad) of the indicated protein band intensities from westernblot analyses of nuclear extracts (Nrf2, Slug) or total lysates (L1, E-cadherin, vimentin) from HPDE cells (**Fig A)** and Colo357 cells (**Fig B)**. Mean values of three independent experiments are shown.
(PDF)

S5 File Densitometric evaluation. Densitometric evaluation (Quantity One software, Bio-Rad) of the indicated protein band intensities from westernblot analyses of total lysates from HPDE cells (**Figs A & C**) and Colo357 cells (**Figs B & D**) cells. Mean values of three independent experiments are shown.
(PDF)

S6 File Potential ARE site in the E-cadherin promoter. **Fig A**) Nucleotide sequence of the human E-Cadherin promoter and the 5‘-end of E-cadherin mRNA (pos. -1357 to +11). The potential ARE site (TGACTCACTA) was identified by screening the nucleotide sequence of the E-cadherin gene (gene bank accession no. DQ090940.1) upstream of the transcriptional start position using the Internet based *Transcription Element Search Software* (http://www.cbil.upenn.edu/tess). The ARE-like sequence is underlined and represents a motif overlapping with an AP1 site. It‘s similarity with the consensus sequence TGACTCAGCA (Malhotra et al. Nucleic Acids Res. 2010; 38(17): 5718–5734) is indicated in bold. **Fig B)** Scheme of the E-cadherin promoter constructs used for luciferase assay either containing (-1189) or lacking (-1153) the ARE site. For comparison, some additional binding sites reported previously (Liu et al., Oncogene. 2005; 24(56):8277–90) were indicated as well, including SP1, E-boxes, acute myeloid leukemia 1 protein (AML1) and hepatocyte nuclear factor 3 (HNF3).
(PDF)

S7 File Densitometric evaluation. Densitometric evaluation (Quantity One software, Bio-Rad) of E-cadherin protein band intensities from westernblot analyses of total lysates from HPDE cells (**Fig A)** and Colo357 cells (**Fig B)**. Mean values of three indpendent experiments are shown.
(PDF)
