# Supplementary figures and images for: The Crosstalk between Nrf2 and TGF-β1 in the Epithelial-Mesenchymal Transition of Pancreatic Duct Epithelial Cells
Source: PLoS One. 2015 Jul 30;10(7):e0132978. doi: 10.1371/journal.pone.0132978 (PMC4520686; doi:10.1371/journal.pone.0132978)

**Fig. A**

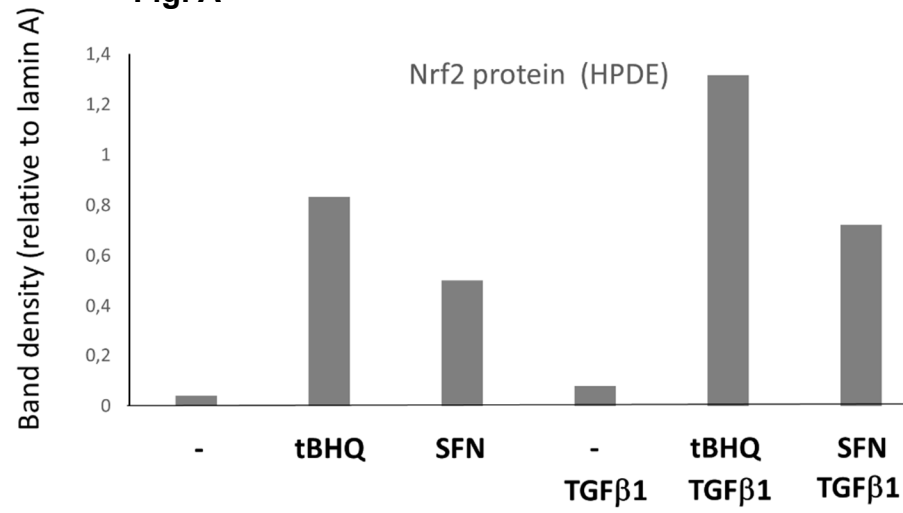

**Fig. B**

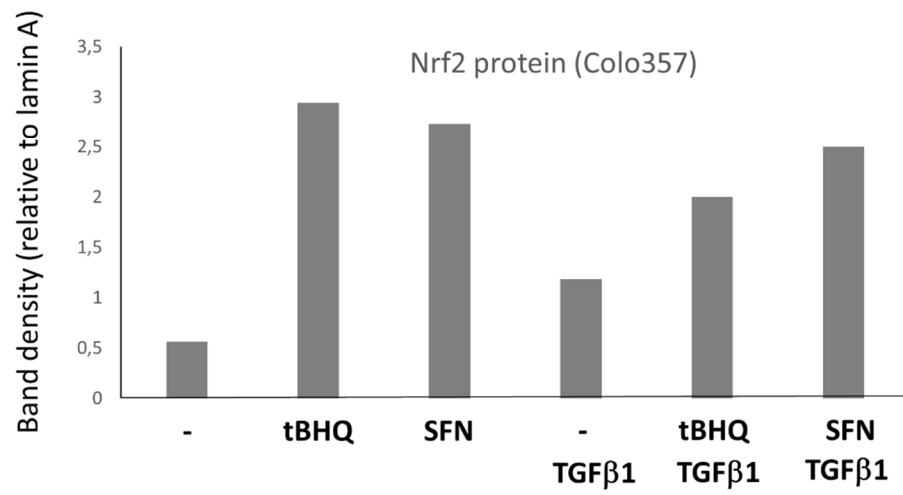

Supplement: S1 File — Densitometric evaluation (Quantity One software, Bio-Rad) of Nrf2 protein band intensities (normalized to LaminA) from westernblot analyses of nuclear extracts from HPDE cells (Fig A) and Colo357 cells (Fig B) cells. Mean values of three independent experiments are shown. (PDF) [file pone.0132978.s001.pdf]

**Fig. A**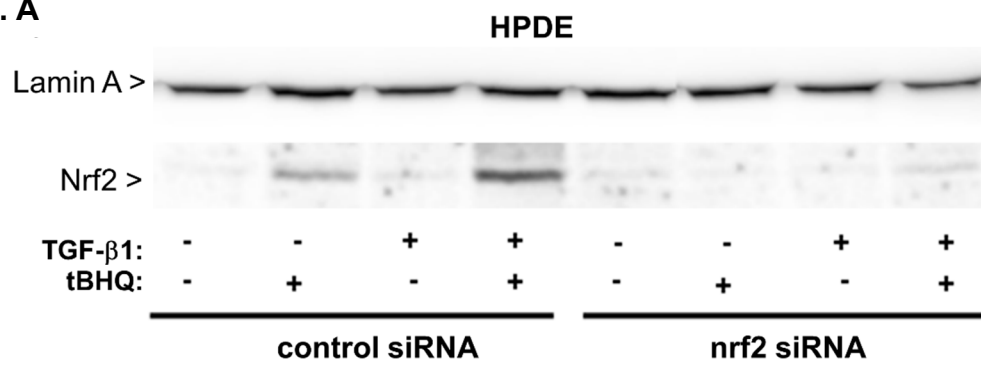**Fig. B**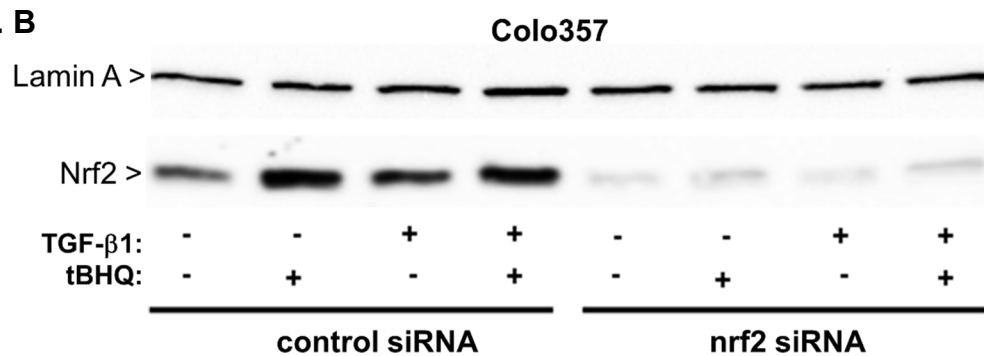

Supplement: S2 File — Nuclear extracts from HPDE cells (Fig A) and Colo357 cells (Fig B) treated with Nrf2 or control siRNA were submitted to Nrf2 westernblot; lamin A was used as loading control. (PDF) [file pone.0132978.s002.pdf]

**Fig. A**

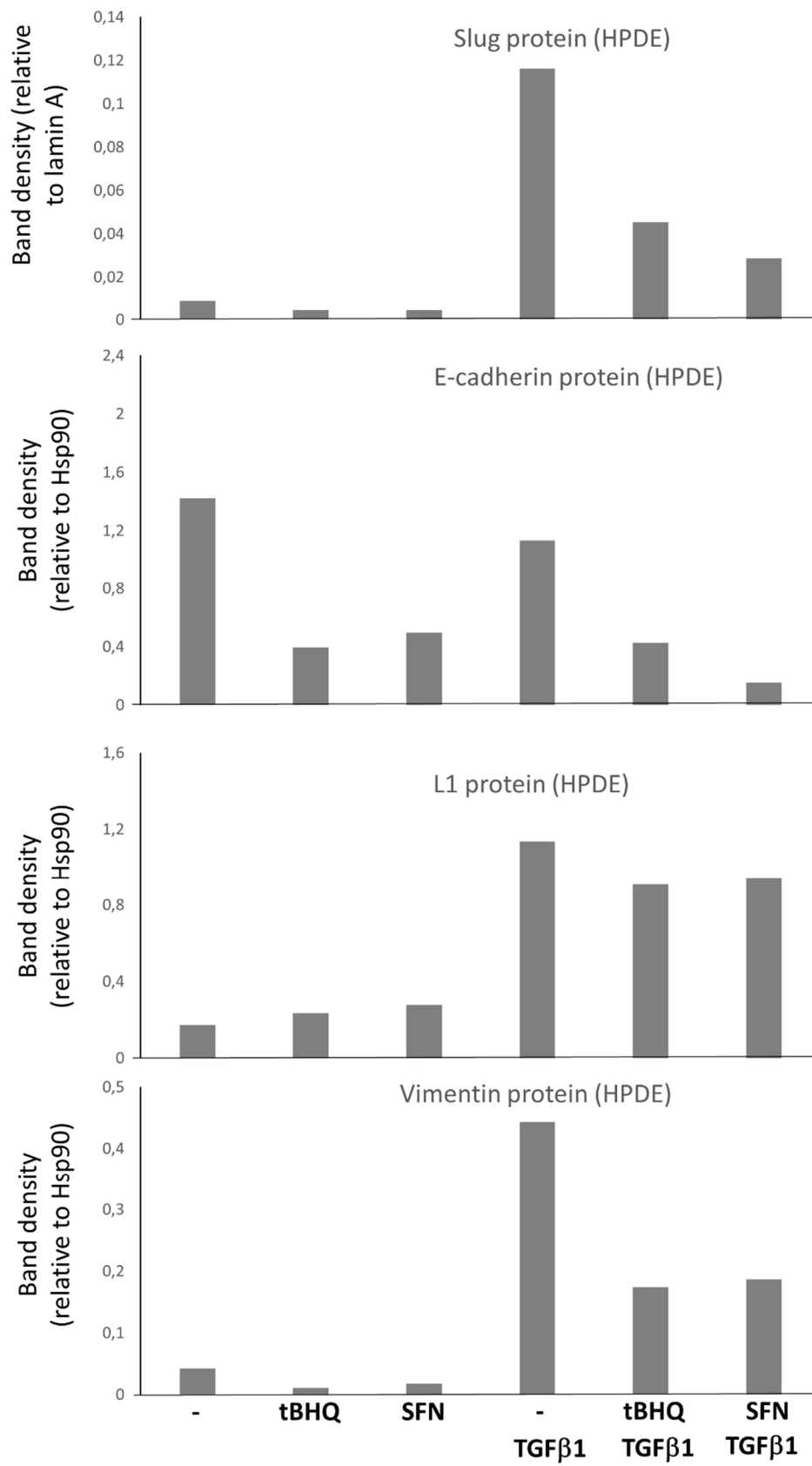

**Fig. B**

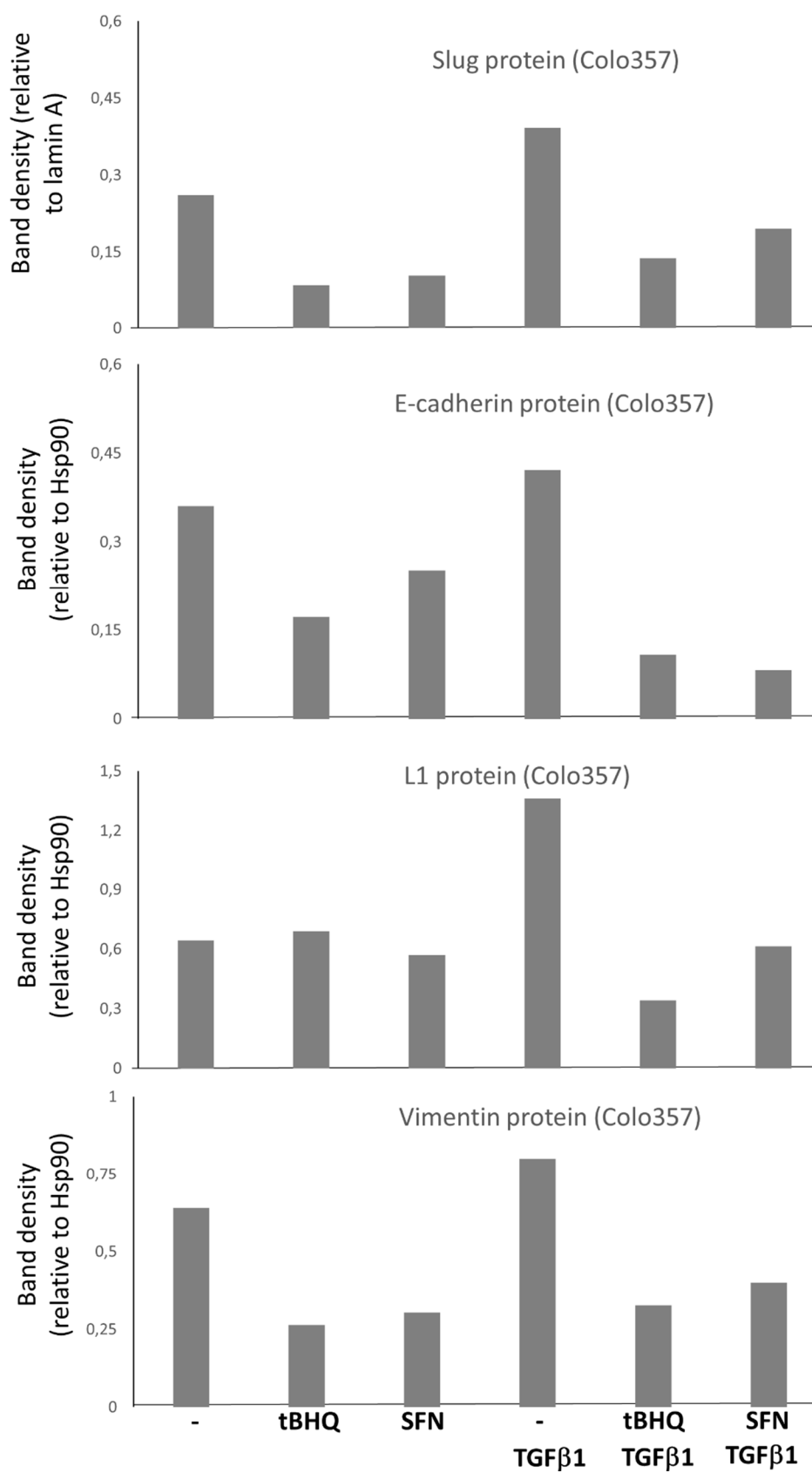

Supplement: S3 File — Densitometric evaluation (Quantity One software, Bio-Rad) of the indicated protein band intensities from westernblot analyses of nuclear extracts (Slug) or total lysates (L1, E-cadherin, vimentin) from HPDE cells (Fig A) and Colo357 cells (Fig B). Mean values of three independent experiments are shown. (PDF) [file pone.0132978.s003.pdf]

**Fig. A**

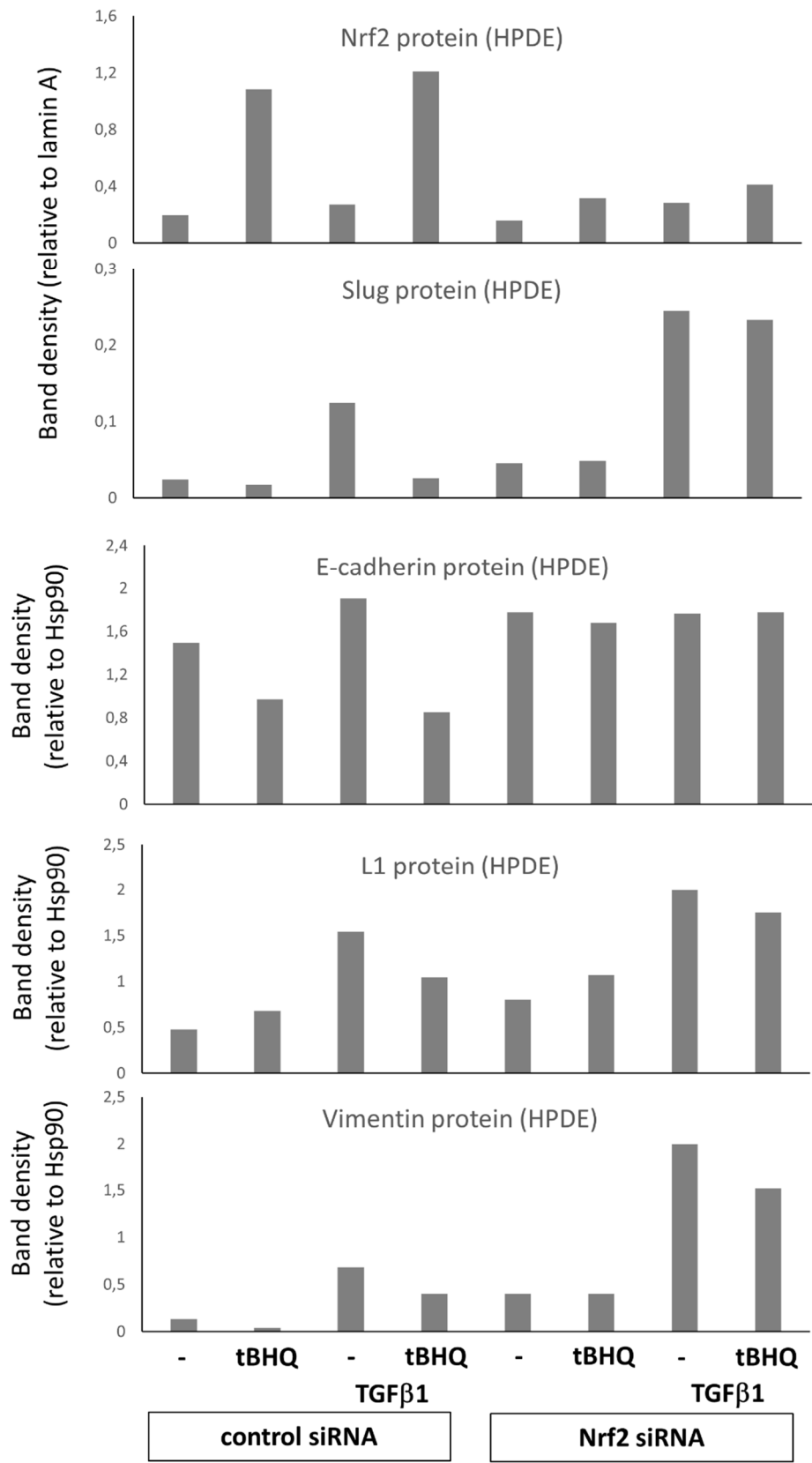

**Fig. B**

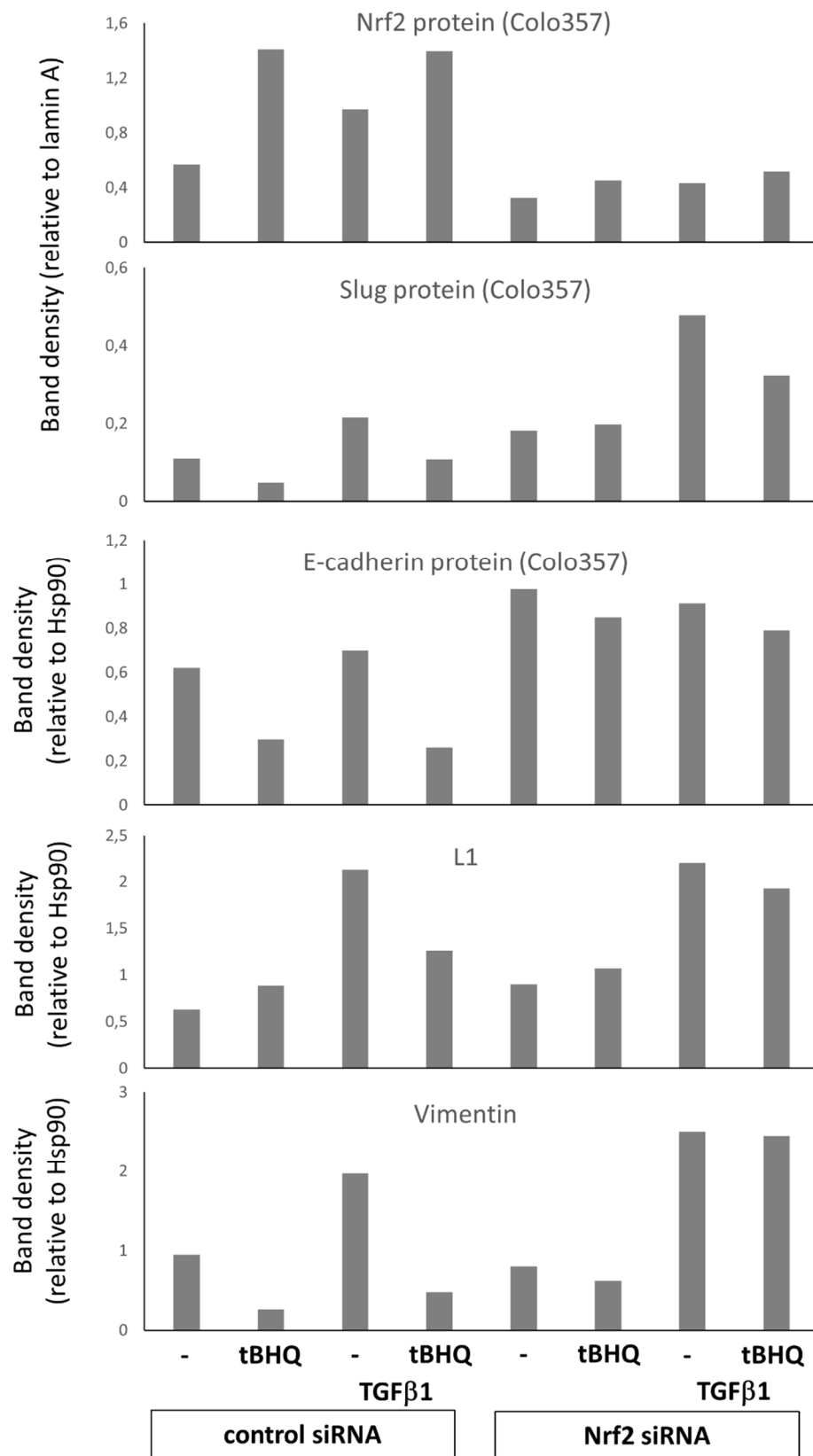

Supplement: S4 File — Densitometric evaluation (Quantity One software, Bio-Rad) of the indicated protein band intensities from westernblot analyses of nuclear extracts (Nrf2, Slug) or total lysates (L1, E-cadherin, vimentin) from HPDE cells (Fig A) and Colo357 cells (Fig B). Mean values of three independent experiments are shown. (PDF) [file pone.0132978.s004.pdf]

**Fig. A**

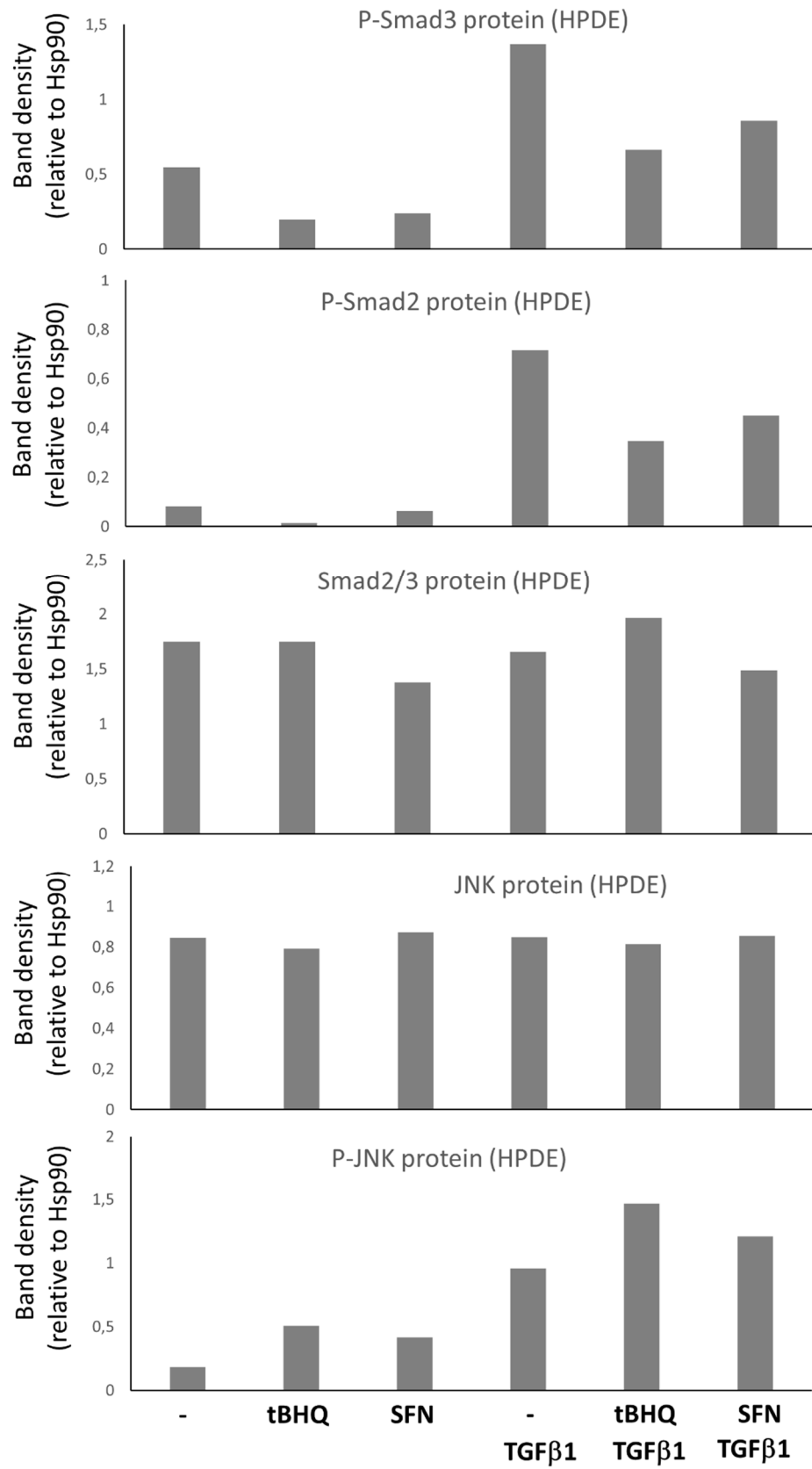

**Fig. B**

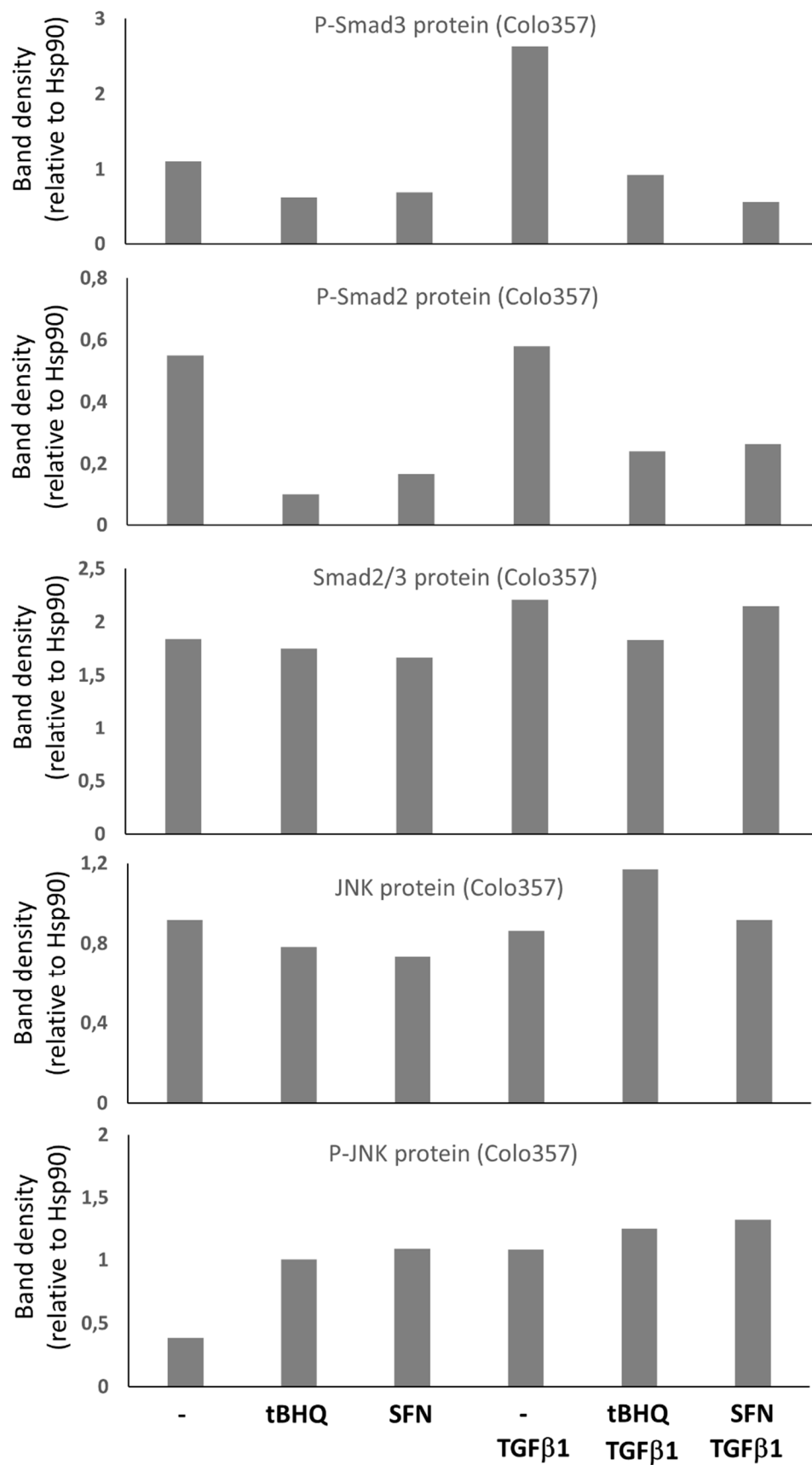

**Fig. C**

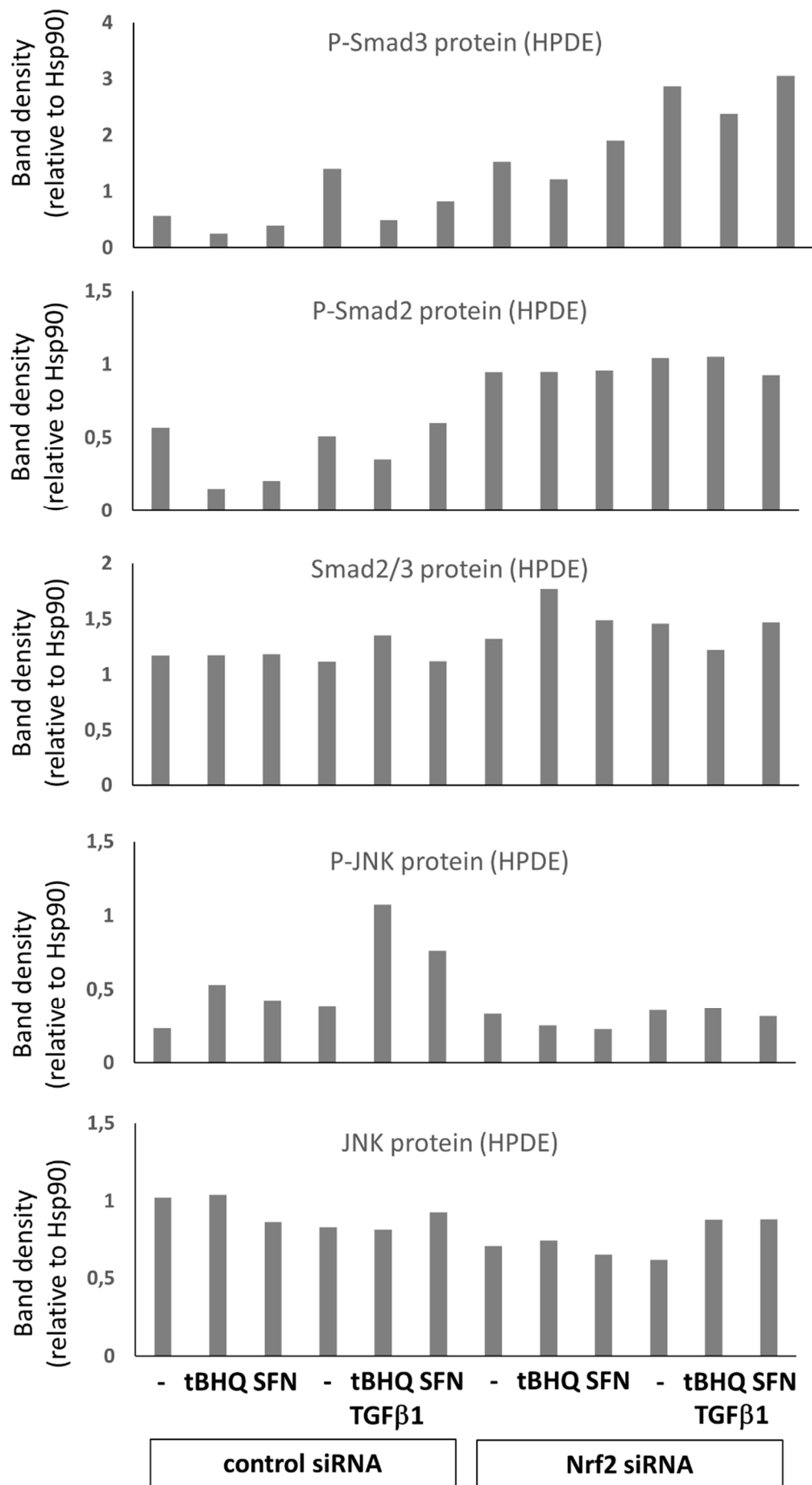

**Fig. D**

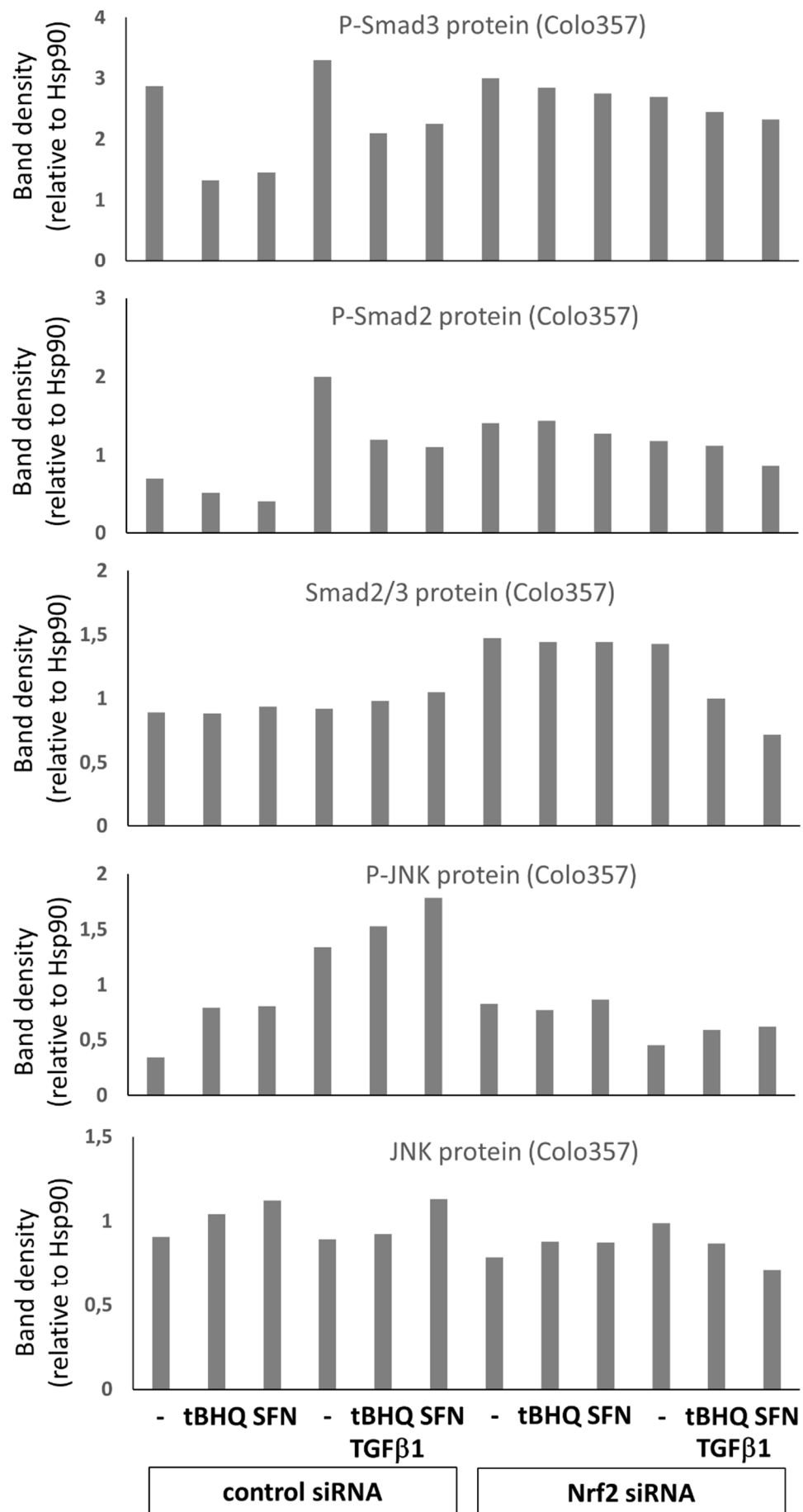

Supplement: S5 File — Densitometric evaluation (Quantity One software, Bio-Rad) of the indicated protein band intensities from westernblot analyses of total lysates from HPDE cells (Figs A & C) and Colo357 cells (Figs B & D) cells. Mean values of three independent experiments are shown. (PDF) [file pone.0132978.s005.pdf]

**Fig. A**

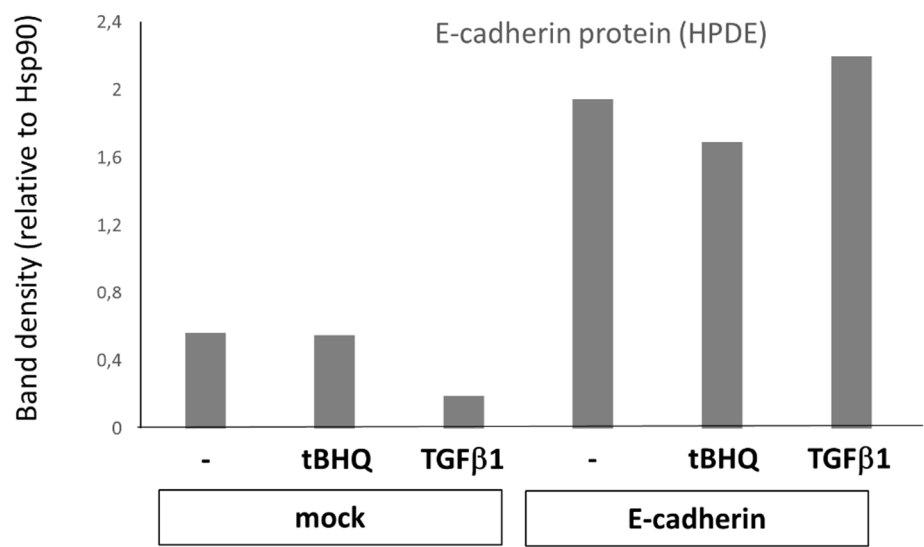

**Fig. B**

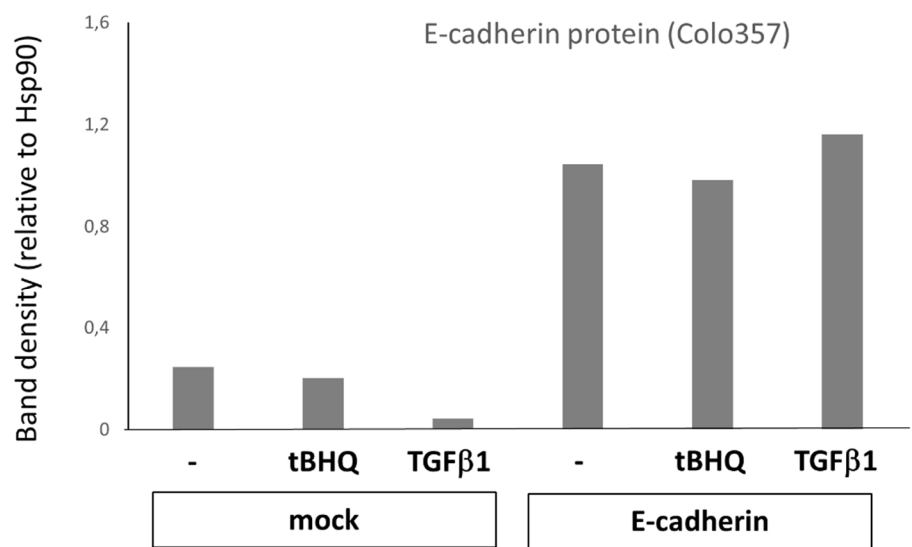

Supplement: S7 File — Densitometric evaluation (Quantity One software, Bio-Rad) of E-cadherin protein band intensities from westernblot analyses of total lysates from HPDE cells (Fig A) and Colo357 cells (Fig B). Mean values of three indpendent experiments are shown. (PDF) [file pone.0132978.s007.pdf]
