# Supplementary material for: The Crosstalk between Nrf2 and TGF-β1 in the Epithelial-Mesenchymal Transition of Pancreatic Duct Epithelial Cells
Source: PLoS One. 2015 Jul 30;10(7):e0132978. doi: 10.1371/journal.pone.0132978 (PMC4520686; doi:10.1371/journal.pone.0132978)
Supplement: S6 File — Fig A) Nucleotide sequence of the human E-Cadherin promoter and the 5‘-end of E-cadherin mRNA (pos. -1357 to +11). The potential ARE site (TGACTCACTA) was identified by screening the nucleotide sequence of the E-cadherin gene (gene bank accession no. DQ090940.1) upstream of the transcriptional start position using the Internet based Transcription Element Search Software (http://www.cbil.upenn.edu/tess). The ARE-like sequence is underlined and represents a motif overlapping with an AP1 site. It‘s similarity with the consensus sequence TGACTCAGCA (Malhotra et al. Nucleic Acids Res. 2010; 38(17): 5718–5734) is indicated in bold. Fig B) Scheme of the E-cadherin promoter constructs used for luciferase assay either containing (-1189) or lacking (-1153) the ARE site. For comparison, some additional binding sites reported previously (Liu et al., Oncogene. 2005; 24(56):8277–90) were indicated as well, including SP1, E-boxes, acute myeloid leukemia 1 protein (AML1) and hepatocyte nuclear factor 3 (HNF3). (PDF) [file pone.0132978.s006.pdf]

Fig. A

E-cadherin promoter

-1357

caccatgcctggccctattgttactatttttacccctcacttctgtacagagca
tttatggctcaagaaacatttgtcattttaattgtatgggagtcccacaacagc
atagggagacatttctgatcattattcccattaggagggtggagaaactgaggc
tttgggaggtggtcctgacctagggaatcaatt<u>**tgctgactcac**</u>taacccatga
agctctacagttaaaaagactagattaaaaatgagaactcagtaaagggct
gaggcaggaggatcgcctgagttcagaaatttgagatcagcctcggcaacatag
tgagatcccctctctagaaaatttttaaaaattaggccgctcgaggcagag
tgcagtggctcacgcctgtaatccaacacttcaggaggctgaagagggtggatc
acctgaggtcaggagttccagaccagcctggccaacatggtgaaaccccgtctg
tactaaaaatacaaaattagccggtgtggtggcacacgcctgtagtcccagcta
ctcaataggctgagacaggagagtctcttgaacccggcaggcggaggttgcagt
gagccgagatcgtgccactgcactccagcctgggcaagacagagcgagactccg
tctcaaaaaatacaaacaaaacaaacaaacaaaaaattaggctgctagctcagt
ggctcatggctcacacctgaaatcctagcactttgggaggccaaggcaggagga
tcgcttcagcccaggagttcgagaccaggctgggcaatacagggagacacagcg
cccccactgcccctgtccgccccgacttgtctctctacaaaaaggcaaaagaaa
aaaaaattagcctggcgtggtggtgtgcacctgtactcccagctactagagagg
ctggggccagaggaccgcttgagcccaggagttcgaggctgcagtgagctgtga
tcgcaccactgcactccagcttgggtgaaagagtgagaccccatctccaaaacg
aacaaacaaaaaatcccaaaaaacaaaagaactcagccaagtgtaaaagccctt
tctgatcccaggtcttagtgagccaccggcggggctgggattcgaacccagtgg
aatcagaaccgtgcaggtcccataacccacctagaccctagcaactccaggcta
gagggtcaccgcgtctatgcgaggccgggtgggcgggccgtcagctccgccctg
gggaggggtccgcgctgctgattggctgtggccggcaggtgaaccctcagccaa
tcagcggtacggggggcggtgcctccggggctcacctggctgcagccacgcacc
ccctctc agtggcgtcggaactgcaaag
+1

Fig. B

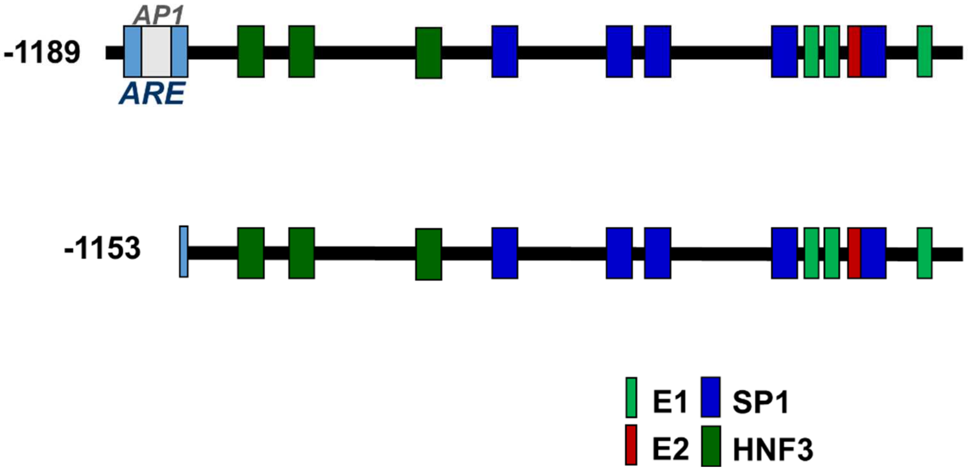

S6 File
